# Factors Influencing the Flavour of Bovine Milk and Cheese from Grass Based versus Non-Grass Based Milk Production Systems

**DOI:** 10.3390/foods7030037

**Published:** 2018-03-13

**Authors:** Kieran N. Kilcawley, Hope Faulkner, Holly J. Clarke, Maurice G. O’Sullivan, Joseph P. Kerry

**Affiliations:** 1 Teagasc Food Research Moorepark, Fermoy, Co., P61 P996 Cork, Ireland; hopefaulkner@hotmail.co.uk (H.F.); Holly.clarke@teagasc.ie (H.J.C.); 2 Department of Nutritional Sciences, University College Cork, T12 R229 Cork, Ireland; maurice.osullivan@ucc.ie (M.G.S.); joe.kerry@ucc.ie (J.P.K.)

**Keywords:** grass, pasture, milk, cheese

## Abstract

There has been a surge in interest in relation to differentiating dairy products derived from pasture versus confined systems. The impact of different forage types on the sensory properties of milk and cheese is complex due to the wide range of on farm and production factors that are potentially involved. The main effect of pasture diet on the sensory properties of bovine milk and cheese is increased yellow intensity correlated to β-carotene content, which is a possible biomarker for pasture derived dairy products. Pasture grazing also influences fat and fatty acid content which has been implicated with texture perception changes in milk and cheese and increased omega-3 fatty acids. Changes in polyunsaturated fatty acids in milk and cheese due to pasture diets has been suggested may increase susceptibility to lipid oxidation but does not seem to be an issue to due increased antioxidants and the reducing environment of cheese. It appears that pasture derived milk and cheese are easier to discern by trained panellists and consumers than milk derived from conserved or concentrate diets. However, milk pasteurization, inclusion of concentrate in pasture diets, cheese ripening time, have all been linked to reducing pasture dietary effects on sensory perception. Sensory evaluation studies of milk and cheese have, in general, found that untrained assessors who best represent consumers appear less able to discriminate sensory differences than trained assessors and that differences in visual and textural attributes are more likely to be realized than flavour attributes. This suggests that sensory differences due to diet are often subtle. Evidence supports the direct transfer of some volatiles via inhalation or ingestion but more so with indirect transfer post rumen metabolism dietary components. The impact of dietary volatiles on sensory perception of milk and dairy products obviously depends upon their concentration and odour activity, however very little quantitative studies have been carried out to date. Some studies have highlighted potential correlation of pasture with enhanced “barny” or “cowy” sensory attributes and subsequently linked these to accumulation of *p*-cresol from the metabolism of β-carotene and aromatic amino acids or possibly isoflavones in the rumen. *p*-Cresol has also been suggested as a potential biomarker for pasture derived dairy products. Other studies have linked terpenes to specific sensory properties in milk and cheese but this only appears to be relevant in milk and cheese derived from unseeded wild pasture where high concentrations accumulate, as their odour threshold is quite high. Toluene also a product of β-carotene metabolism has been identified as a potential biomarker for pasture derived dairy products but it has little impact on sensory perception due to its high odour threshold. Dimethyl sulfone has been linked to pasture diets and could influence sensory perception as its odour threshold is low. Other studies have linked the presence of maize and legumes (clover) in silage with adverse sensory impacts in milk and cheese. Considerably more research is required to define key dietary related impacts on the flavour of milk and cheese.

## 1. Introduction

Recent years have seen a significant rise in interest regarding dairy products produced from pasture or grazing, mainly because such products are purported to have “added value” for the consumer [1,2,3,4,5]. This is mostly due to a rise in consumer awareness with regard to; health benefits (omega-3 polyunsaturated fatty acids and conjugated linoleic acid) of dairy fat, food traceability/authentication, food and environmental sustainability, animal-welfare (less intensive farming), local origin of food production (support local business, lower transport costs), terroir (geographical origin status) and organic production [6,7,8,9,10,11,12,13,14].

One aspect of the effect of ruminant diet on the quality of bovine milk and cheese that remains somewhat ambiguous is the impact of diet on sensory perception (including taste, aroma, texture and colour). Obviously this is an important aspect as sensory properties are well recognized as prime purchasing factors [15] but also because milk and dairy products from grazing are often perceived as higher quality and can achieve a higher price at farm and retail level [10,13]. The impact of diet on sensory perception of bovine milk and dairy products is unclear because of the fact that a wide range of potential factors are involved at farm level (e.g., type of forage, grassland management, forage conservation methods/practices, animal breeds, stage of lactation, health status of the animal) and during production (e.g., heat-treatment, product type, storage fermentation). Factors that potentially influence the flavour of bovine milk and cheese that derive from diet, include compounds that can potentially transfer directly from forage into milk (and subsequently into cheese produced thereof) or compounds which act as substrates for compounds that eventually culminate in milk or cheese.

A significant amount of studies investigating the sensory aspects of bovine milk and dairy products resulting from ruminant pasture/grazing have been undertaken but have not been recently reviewed [2,16,17]. This review focusses on the potential impact of pasture/grazing in comparison to confined feeding systems and the use of preserved forages or concentrates on the sensory perception of bovine milk and cheese.

## 2. Impact of Forage on the Fatty Acid Content of Bovine Milk and Cheese

It is well established that diet influences the fatty acid (FA) profile of milk with pasture feeding increasing the levels of health beneficial polyunsaturated fatty acids (PUFA), namely the omega-3 FA in comparison to confined feeding systems [13,18,19,20,21,22]. The most important PUFA in terms of health promoting aspects in bovine milk are conjugated linoleic acid (*c*9, *t*11 CLA), eicosapentaenoic (EPA) and docosapentaenoic (DPA) acids [10,14,21,22,23]. All FA with 18 or more carbons originate from ingested feed [13] with PUFA composition and concentration predominately influenced by intake of long chain PUFA, the precursors of which are linoleic acid (LA) and α-Linolenic acid (ALA). LA and ALA are present in higher concentrations in fresh pasture than in concentrates [24]. Ruminal bio-hydration of LA and ALA leads to formation of vaccenic acid (VA), the precursor of CLA in the mammary gland [24]. Thus, VA is also higher in milk from pasture [13]. Ingested FA are also bio-hydrogenated and isomerized in the rumen and desaturated with Δ9-desaturase in the adipose tissue and mammary gland, altering the FA profile. PUFA in the bovine diet are extensively bio-hydrogenated in the rumen, if unprotected about 70–95% LA and 85–100% of linolenic acid (LNA) are bio-hydrogenated in the rumen [20]. Shorter chain fatty acids (SCFA), C15:0 and less are primarily produced by de novo synthesis in the mammary gland using fatty acid synthase and acetyl-coenzyme (A carboxylase) and are also impacted by genetic factors [25]. Palmitic acid (C16:0) is formed by both de-novo synthesis and directly from feed. The extent and completion of the bio-hydrogenation process depends on rumen conditions, diet composition and is inhibited by large amounts of LA [6]. Ruminal bio-hydration intermediates also act as regulators of disruptors of mammary lipogenesis which impact on the amount of secreted milk fat but also on FA composition, including SCFA. Thus ruminal bio-hydration modifies yields and/or interacts with all milk FA and plays an important role in the interaction between ruminant diet and mammary FA synthesis and secretion [19]. Grazing pasture reduces de-novo synthesis in the mammary gland due to intake of longer chain FA, such as ALA. The impact of forage on the production of SCFA is less important as they are de-novo synthesized but as mentioned long chain FA such as ALA can influence the process. In free form SCFA resulting from hydrolysis of dairy fat; especially butanoic, hexanoic and octanoic acids are very important flavour compounds in many cheese varieties, partly because they are relatively easily hydrolysed due to their position on the glyceride backbone (mainly sn-3) and their water solubility and odour activity [26]. These free SCFA are described as having a cheesy aroma [27] but are also described as rancid (butanoic acid), sweaty (butanoic acid) and goaty (hexanoic and octanoic acids), which can be perceived as positive or negative depending upon the cheese variety and their concentration. These SCFA are also substrates for a range of flavour compounds in milk and fermented dairy products; esters, ketones, aldehydes and alcohols [26].

Longer chain FA do not have a major direct impact on the flavour of milk and dairy products but may have an important indirect role in flavour as these long chain FA and can be hydrated, oxidized, or reduced to odour active aldehydes, ketones, lactones and alcohols [28,29,30]. Increasing levels of unsaturation in FA as evident in pasture grazing may increase the susceptibility of oxidation, despite the presence of natural anti-oxidants (tocopherols, vitamins, lactoferrin, enzymes, carotenoids and urate) in the milk [5,9,31]. A study by Hedegaard et al. [32] found that bovine milk with increased PUFA was more susceptible to oxidation. Thus, arachidonic acid (AA) and docosahexaenoic acid (DHA) are more susceptible to oxidation than LA but the abundance of individual PUFA in milk must also be taken into account [33] as levels of AA and DHA are very low in comparison to palmitic and oleic acids. Bendall [34] suggested that oxidation of LNA may be more important for flavour than oxidation of either LA or oleic acid as oxidation occurs more rapidly with increasing level of unsaturation. Some of the short straight chain aldehydes, especially hexanal, propanal, heptanal and pentanal are widely used as indicators of lipid oxidation [31,33,35] in dairy products. For example, hexanal mostly results from the degradation of oleic acid and LA [36] which are present in higher concentrations in milk derived from pasture than from confined systems [10,13,37]. Pentanal is derived mainly from the oxidation of AA and LA [28,33], which are generally higher in bovine milk from pasture grazing [22,28]. Both hexanal and pentanal are characterized by a cardboard-like, or metallic-like off-flavour [30]. These aldehydes are relatively transient compounds in dairy products, especially in cheese where they are readily reduced to alcohols and acids [35]. Primary alcohols are derived from their respective aldehydes, thus increased levels of hexanal and pentanal in milk and dairy products will likely also result in increased levels of 1-hexanol and 1-pentanol. A wide range of straight and branched chain ketones have also been implicated in lipid oxidation with both 3-Octen-2-one and 3,5-Octadien-2-one generated from oxidation of LA and AA contributing to a mushroom odour [29]. These ketones are potentially important as LA and AA tend to be at higher concentrations in milk from pasture grazing [10,22]. In the rumen C18 unsaturated FA are transformed by hydration into hydroxy acid intermediates. After absorption the hydroxy acids are shortened by three rounds of β-oxidation followed by cyclization to γ-lactones in animal tissue [28]. For example oleic acid and LA are precursors of flavoursome γ-dodecalactone and γ-dodecaenolactone, respectively [28] and thus levels could potentially be higher in milk from pasture grazing. As mentioned oxidation in milk is controlled to an extent by the presence of antioxidants (enzymes, tocopherols, vitamins, lactoferrin, carotenoids and urate) [9], which are often higher in milk derived from pasture [14,20,22,38,39,40]. A study by Faulkner et al. [41] did not find any sensory perception differences related to lipid oxidation in bovine milk derived from either pasture (rye-grass), pasture (rye-grass) including white clover or concentrate (grass silage, maize silage, maize, beet pulp, soybean meal, salt, rapeseed meal, megalac) despite differences in FA profiles [22]. This indicates that the antioxidant level may counteract high levels of PUFA in pasture derived milk. The impact of lipid oxidation in cheese is less important than milk as cheese is a very reducing environment. Some key volatiles associated with lipid oxidation that may impact on milk and cheese is listed in Table 1.

The FA profile can also influence the texture of milk and dairy products. The degree of unsaturation, FA chain length and the FA position on the triacylglycerol influences the melting point of fat and thus can affect the texture of milk and dairy products derived from that milk [2,41]. Palmitic and oleic acids are the principal saturated and unsaturated FA in dairy products with high (62.9 °C) and low (13–14 °C) melting points, respectively [28]. The ratio of oleic acid to palmitic acid has previously been used as an index of hardness in butter and cheese [2,42]. In a study by Villeneuve et al. [28] the ratio of palmitic acid to oleic acid was lower in milk from cows on pasture than those fed hay or silage. A lower ratio of oleic to palmitic acid is associated with a decrease in the perception of firmness and an increase in melting in the mouth [42,43,44]. Two studies found viscosity changes in milk due to diet [26,28]. Faulkner et al. [41] found a greater perception of viscosity in milk from rye-grass pasture in comparison to milk derived from concentrate (grass silage, maize silage, maize, beet pulp, soybean meal, salt, rapeseed meal, megalac) which corresponded with a lower ratio of oleic acid to palmitic acid [22]. Moorby et al. [45] found that milk derived from grass silage was more viscous than milk derived from silage with a higher concentration of red clover.

In summary pasture diets increase certain PUFA levels in milk, especially the concentration of healthy omega-3 FA. However, the susceptibility to lipid oxidation may be increased, although this does not seem to be an issue in milk due the presence of anti-oxidants or in cheese due to the reducing environment but may be an issue in whole milk powders or butter. Pasture feeding also changes the ratio of palmitic to oleic acids and thus can alter the texture or viscosity perception of resultant dairy products.

## 3. Impact of Carotenoids (β-Carotene and Lutein) on the Sensory Perception of Bovine Milk and Milk Products

Carotenoids are potential biomarkers as both lutein and β-carotene are at their highest concentrations in dairy products derived from pasture [39]. Carotenoids in bovine milk and in milk products are important for human health and nutrition as well as been natural antioxidants some are also precursors of vitamin A, with all-*trans*-β-carotene been the main pro-vitamin [40]. The β-carotene concentration of bovine milk is impacted by the nature and amount of forage intake as well as transfer to the mammary gland. In a study of farm milks, Agabriel et al. [46] found that β-carotene concentrations varied in milk by ~50% between early and late season. Rumen digestion, intestinal absorption and tissue metabolism are also important factors that govern carotenoid levels in milk. Ruminal degradation of β-carotene varies depending upon dietary source. The colour of dairy products is highly dependent upon the concentration of carotenoids, especially β-carotene [39]. β-carotene concentration is much higher in ruminants with only bovines accumulating high concentrations and none in ovine or caprine milk [39]. This is a main factor why caprine and ovine milks are whiter than bovine milk. Milk carotenoids are transferred into butter and cheese with minimal losses as they are fat soluble [39]. Cartenoid content is reduced considerably in conserved forages mainly due to exposure to ultra-violet rays which is more extensive for β-carotene than lutein. Wilting and ensiling processes also decrease carotenoid content and are dependent upon content and pH [39]. Prache et al. [47] stated that carotenoid content is decreased by ~60% for dehydrated forage and wilted silage, 70% in barn-dried hay and 80–90% for field-cured hay in relation to levels in fresh forage and also decreases with conservation duration.

The yellow colour of dairy products has positive associations with traditional farm practices by the consumer and yellow coloration of dairy products is generally more important for high fat products such as butter and some full fat cheese [39]. The impact of pasture and conserved forages on the colour of cheese has been widely reported [3,22,48,49,50] and in milk [26]. However, in some studies consumers did not perceive differences in milk colour from cows fed pasture, pasture plus supplements (grain and minerals) or concentrate (alfalfa hay, corn silage, seam-flaked corn, grain, cottonseed, soybean meal, sugar beet pulp, yeast mix, molasses and fat) [51]. Moorby et al. [45] also found that milk became whiter as the portion of red clover in grass/clover silage diet increased which is in agreement with β-carotene loss [39,47]. As mentioned the main antioxidants in milk are enzymes, lactoferrin, tocopherols, vitamins C and E, urate and carotenoids. Cartenoids function as singlet oxygen scavengers and β-carotene has an important role in the prevention of photo-oxidation as it absorbs light in a concentration dependent manner that would otherwise be absorbed by riboflavin. This implies that milk from pasture feeding maybe less susceptible to light oxidation. It is difficult to determine the exact anti-oxidant effect of β-carotene alone as pasture rich milks also contain high levels of other anti-oxidants. Some authors have suggested that β-carotene could be a suitable biomarker to distinguish pasture produced dairy products from other diets [22,39,41,52], but further work is required to set specific product concentrations as β-carotene is also present in conserved feed and concentrate albeit at lower levels. Oxidation is not a problem in cheese because of the reducing environment. However, metabolism of β-carotene has also been correlated to increased levels of *p*-Cresol and toluene in milk and cheese [41,52] (Table 1) (covered in more detail in Section 5).

In summary β-carotene levels in dairy products are positively correlated with yellow colour. β-carotene is a potential biomarker for pasture derived dairy products due to its higher concentration but specific levels would have to be determined for each dairy product as β-carotene is present in other dietary sources, such as conserved diets but at lower levels due to Ultraviolet (UV) exposure. Pasture derived dairy products may also be less susceptible to photo-oxidation due to higher concentrations of β-carotene.

## 4. The Potential Impact of Volatile Terpenoid Compounds Derived from Diet on the Sensory Perception of Bovine Milk and Cheese

Volatile compounds that may directly influence flavour can either be absorbed in the digestive tract, i.e., rumen and or intestine before diffusing into the blood and then transferred to the mammary gland or by the pulmonary route, where volatiles are inhaled into the lungs, then enter the blood stream and subsequently diffuse into the mammary gland [53]. Terpenoids (terpenes) are a group of plant secondary metabolites derived from isoprene units (C5), monoterpenes (C10) and sesquiterpenes (C15) [40]. The composition of these compounds in milk is directly related to the composition of the same compounds in the feed [53,54]. Terpenes (monoterpenes and sesquiterpenes) are odour active but have a high odour threshold. However, they have been reported to have a role in the flavour of some milk and cheese products [55,56,57]. Milk obtained from animals grazing pasture has been suggested to have a much greater diversity of terpenes than milk from confined systems (preserved forages or concentrates) [2,54,58]. It appears that the transfer of terpenes from forage into the milk is relatively rapid, 8 h for mono-terpenes and 32 h for sesquiterpenes [53]. Lejonklev et al. [59] found that both oral and gastrointestinal introduction occurred within 9 h in bovine milk. However, it also appears that if terpenes are removed from the diet they can disappear from the milk within a few days [53,59]. This highlights a potential fluctuating impact of terpenes in milk and dairy products, especially when derived from wild diverse pastures.

Terpenes in grassland plants vary widely according to botanical family with dicotyledones typically containing more than monocotyledons [4,60]. This is nicely proven by the study of Agabriel et al. [46] who found that the terpene content in milk and cheeses were higher if cows were fed natural dicotyledon-rich pastures rather than monospecific forage or concentrates. Fernandez et al. [58] and Engel et al. [61] also found a higher sesquiterpene/monoterpene ratio in highland forage than in lowland grazed pasture. Fernandez et al. [58] identified six sesquiterpenes (δ-elemene, β-bourbonene, β-caryophyllene, β-chamigrene, γ-cadinene and β-sesquiphellandrene) that fully discriminated milk from highland, lowland, pasture and indoor (concentrate) feeding. Tornambé et al. [60] found that β-cariphyllene and α-copaene were the most abundant sesquiterpenes in milk samples. Bugaud et al. [54] found the greatest number of monoterpenes (30) and sesquiterpenes (35) in bovine milk from diversified pastures and found correlation between monoterpenes in the milk and in the pasture (terpinolene, tricyclene, β-Pinene, α-fenchene, camphene, limonene, α-terpinene, α-pinene, β-mycrene, α-phellandrene, *p*-cymene and γ-terpinene). Highest levels of terpenes were found in mountain milks and lowest in lowland milks on a diet of hay. Only two sesquiterpenes identified in forage were found in milk—cloven and α-cedrene. Viallon et al. [62] compared terpenes and sesquiterpenes in St-Nectaire-type cheeses made from cows milk derived from different hay diets (cocksfoot, rye-grass and native mountain pasture). They found that hay from native mountain pasture contained more terpenes and sesquiterpenes than rye-grass hay with the lowest amount in cocksfoot hay. The most abundant terpenes and sesquiterpenes (limonene, β-phellandrene, *p*-cymene, β-pinene and α-pinene) were found in forages were also the most abundant in the cheeses made from milks derived from these same forages. Carpino et al. [63] also found higher levels of terpenes (citronellol and carvone) in Ragusano cheese produced from the milk of cows fed native pasture than from milk from cows fed concentrate (based on maize silage and rye-grass). This study also proved that volatile compounds from pasture could be transferred to milk and subsequently into cheese. De Noni and Battelli [64] found the terpenoid profile of milk samples produced from mountain pastures mainly consisted of the monoterpenes (α-pinene, β-pinene, β-mircene, sabinene, camphene, δ-3-carene and limonene) and these were similar for ripened Bitto cheese produced from these same milks. The lack of potential terpenes in monocultures seem to be supported by studies on milk, cheese and butter produced from monocultures of perennial rye-grass and perennial rye-grass seeded with white clover where very little terpenes were identified [22,65]. Coppa et al. [57] also found that diverse pasture derived milk had significantly (*p* < 0.05) more monoterpenes (camphene and sabinene) and a sesquiterpene (β-caryophyllene) than milk from a hay diet, they also noted that milk from cows which continuously grazed pasture had more sesquiterpenes (β-caryophyllene, alloaromadendrene, d-germacrene and γ-cadinene) and monoterpenes (β-pinene and *p*-cymene) than milk from cows on rotational grazing pasture and from hay, suggesting possible selection by cows if forage is in abundance. Tornambé et al. [60] found similar results, they evaluated terpenes in milk from cows on stripped grazing and paddock grazing and found that the most abundant monoterpenes in milk were β-pinene, α-pinene, γ-terpinene, limonene, α-tujene, terpinolene and α-phellandrene and the most abundant sesquiterpenes were β-caryophyllene, α-copaene, β-cedrene, *trans*-4-(14)-5-diene, β-bisabolene and γ-cadinene. These authors also identified that grazing management impacted on terpene levels in milk, suggesting that cows appear to select certain forage.

Agabriel et al. [46] found that the concentration of monoterpenes was greater than sesquiterpenes in milk with α-pinene, β-pinene, limonene and p-mentha-1,8-diene present at the greatest concentrations. These authors also found that terpene levels increased markedly from winter milk (maize silage) to summer pasture-based grazing. A study by Chion et al. [66] found similar trends in milk with α-pinene, β-pinene and δ-3-carene dominating. Toso et al. [67] found that the total concentration of α-pinene, limonene and *p*-cymene was greatest in milk from cows fed diets of hay (lucerne), hay (lucerne) and maize silage and lower in milk from maize silage and lowest in milk from grass (lucerne) silage. Engel et al. [61] suggested that loss of monoterpenes is greater during wilting than sesquiterpenes due to their higher volatility. It is also possible that lower levels of terpenes in silage are due to bioconversion by microorganisms in the fermentation process [40,68]. However, Boltar et al. [69] found that terpenes (α-pinene and *p*-cymene) were higher in feed and in bovine milk produced from grass silage in winter than from pasture (no details provided) in the summer. They also identified five monoterpenes (pinane, α-pinene, sabinene, limonene and *p*-cymene) and a sesquiterpene (β-caryphyllene) in Nanos cheese from summer and winter milks. Higher amounts were present in summer Nanos cheese with only sabinene not detected in winter Nanos cheese. These authors also highlighted a potential direct transfer between forage and milk for pinane and sabinene. Bovolenta et al. [70] found 7 monoterpenes (α-pinene, camphene, β-pinene, sabinene, limonene, 1,8-cineol and 8-hydroxylinalool) and a sesquiterpene (caryophyllene) in Montasio cheeses produced from nutrient rich (poaceae, cyperaceae, ranunculaceae and fabaceae) and poor (poaceae, cyperaceae, asteraceae fabaceae and rosaceae) pastures. α-Pinene, limonene, camphene and 1-8-cineol were significant higher in cheeses made from nutrient poor pasture.

Limonene is often widely present in milk and was the most common terpene in milk from a range of highland and lowland pastures (rye-grass, clover) or in concentrates (maize silage, hay, cereals) in different seasons [58]. Toso et al. [67] also found that limonene was the most abundant terpene in milk from hay (lucerne), maize silage and grass (lucerne) silage. Tornambé et al. [60] also found that most terpenes varied in milk but that limonene was present at high levels in all milk samples, similar to that found by Viallon et al. [62]. Buchin et al. [71] suggested that limonene is prevalent in milk and dairy products because it is found in a wide variety of plants. Limonene has also been seen to accumulate in cheese over ripening suggesting that it may also be a product of bioconversion of sesquiterpenes [52]. Moio et al. [55] suggested that terpenes undergo little bacterial modification in the rumen and noted that sesquiterpenes had only a limited effect on odour and postulated that sesquiterpenes were absorbed via the gastrointestinal tract where terpenes were more likely inhaled and absorbed. However, Poulopoulou et al. [72] highlighted that biotransformation of terpenes in ruminants may occur both at the rumen and in the lower intestine and that removal or detoxification by hepatic or urinary routes may occur when the concentration rises above a certain threshold. Bugaud et al. [54] also noted that none of the oxygenated monoterpenes quantified in pasture (gramineae, apiaceae and geraniaceae) were found in milks, suggesting that they may be easily metabolized by rumen bacteria. Schlichtherle-Cerny et al. [73] incubated rumen fluid with a specific plant (Heracleum sphondylium) and quantified levels of terpenes before and after incubation. These authors noted that out of 20 terpenes identified all were reduced over a 12 h incubation. (*Z*)-β-Ocimene, (+)-β-pinene were completely metabolized, with β-mycene, (*E*)-β-Ocimene, δ-3-carene almost fully metabolized. In addition, five new terpenes were formed (3,7-dimethyl-1,6-octadiene; menth-1-ene; 3,7-dimethyl-2-octene; 2,6-dimethyl-2,6-octadiene and camphene). This study highlights the potential for terpenes to be bio-synthesized and metabolized (hydrogenated) in the rumen. Belviso et al. [74] highlighted that lactic acid bacteria can modify and bio-synthesize terpenes and thus terpene content may be modified in the rumen and in milk or cheese during ripening. This was further supported by the fact that these authors also found some of the same terpenes in forage and in milk and in Raclette-type cheese derived from the same forage as well as additional terpenes in cheese and milk that were not present in the original forage.

Some studies did not find any difference in terpene content in cheese based on diet. Verdier-Metz et al. [48] produced St-Nectaire type cheeses from milk produced from cows fed either grass silage or hay from the same sward but did not find any significant differences in terpenes between the cheeses. Similarly Stefanon and Procida [75] did not find any differences in α-pinene and limonene content in Montasio cheeses in milk from cows fed three distinct diets; lucerne hay, lucerne hay and maize silage and from lucerne hay, maize silage and grass silage. Chion et al. [66] found monoterpenes (limonene, α-pinene, camphene, β-pinene, δ-3-carene, β-mycene and sabinene) in milk samples from cows in winter which were indoors and fed hay (poaceae, fabaceae, lamiaceae and aseraceae) with concentrate (corn grain, soybean meal, corn gluten feed, wheat bran, sunflower meal, rich hull, calcium carbonate, molasses and minerals) and in summer fed natural grassland pasture (poaceae, fabaceae, lamiaceae and aseraceae). Summer milk was richer in terpene content with α-pinene and β-pinene the most abundant terpenes. Only negligible levels of sesquiterpenes were apparent. The terpene content of Toma Piemontese cheese produced from winter and summer milk followed a similar pattern. Also a study by Cornu et al. [44] on Cantal-type cheese made from raw and pasteurized milk from pasture (no details provided) and concentrate (flattened barley and soybean meal) using gas chromatography olfactometry noted that several terpenes were identified in the cheese but none were perceived as odour active.

Overall as terpenes increase as plants mature and fructify [60] and as significant variations exist in forage due to botanical diversity, geographical location (soil/environment) and grassland management, preservation methods, presence in feeding supplements and the fact that they appear to be biosynthesized and metabolized in the rumen and in cheese over ripening and it is very unlikely that they are useful indicators or biomarkers for dairy products from grazing. However, in some circumstances such as for milk or dairy products resulting from unique forage in specific alpine regions, where dicotyledones are abundant they can potentially influence sensory perception due to high concentrations. As their odour threshold is high (possibly more so for sesquiterpenes than monoterpenes), it may be assumed that they would need to be present at very high concentrations to have a sensory impact. Thus, it is more likely that they can impact the sensory perception of milk or fresh cheese rather than mature cheeses. Evidence also exits for possible direct transfer from forage into milk but more research is required to confirm this. Some common terpenes found in milk and cheeses are listed in Table 1.

## 5. Volatile Compounds from Diet Influencing the Sensory Properties of Bovine Milk and Cheese

Several groups of non-terpenoid volatile compounds are believed to contribute to the flavour of milk; such as esters, acids, lactones, phenolics, sulphur compounds, aldehydes and alcohols. However, cheese flavour is much more complex due to the activity of high numbers of added microbes (mainly lactic acid bacteria, yeasts and moulds) and therefore even though similar chemical classes of compounds exist in cheese they are usually present at significantly higher concentrations due to the on-going fermentation processes over ripening. As a significant range of non-terpene volatiles are produced by fermentation in cheese, it makes it more difficult to determine if diet was a factor in their formation. Bendall [67] and Croissant et al. [3] found similar volatiles in milk from pasture and from pasture plus concentrate or from concentrate diets, except that relative abundance correlated with diet, suggesting that it is not presence or absence but abundance that is more important. As discussed earlier, the FA profile of milk is influenced by diet and as stated higher levels of PUFA in milk derived from pasture grazing, may result in increased oxidation in milk and subsequent dairy products. Havemose et al. [31] attributed the amount of ketones to the amount PUFA in milk and also postulated that increased levels of LNA in milk from cows on grass/clover diets were a significant factor in the development of lipid hydroperoxides, aldehydes, ketones and alcohols arising from lipid oxidation. Cornu et al. [44] investigated the impact of pasture (no details provided) and concentrate (flattened barley and soybean meal) on the volatile profile of Cantal type cheese made from raw and pasteurized bovine milk. Volatiles did not appear to be impacted by diet but were significantly impacted by heat-treatment (raw vs. pasteurized) or ripening time (3 months vs. 6 months). Indicating that pasteurization and ripening time had greater significant effects on cheese volatiles than diet.

### 5.1. Aldehydes

Villeneuve et al. [28] suggested a possible link between pentanal and intensity of grassy flavour in milk derived from pasture (timothy) which may be due to the oxidation of AA and LA which is higher in pasture derived milk [28,33]. This same association was also noted by Faulkner et al. [41] who found that levels of pentanal were higher in milks derived from pasture (rye-grass) than from concentrate (grass silage, maize silage, maize, beet pulp, soybean meal, salt, rapeseed meal and megalac). These authors also noted that 1-pentanol was also higher in these same milks and most likely arose from the reduction of pentanal. They also found that hexanal, a product of the degradation of oleic acid and LA was present at higher levels in milk derived from concentrate in comparison to milk derived from the pasture diets and this corresponded well with higher levels of oleic and LA in the milk from cows fed concentrate. However, in this study neither aldehyde appeared to impact on the sensory perception of milk, presumably as the concentrations were lower than their aroma thresholds. Carpino et al. [63] found a significant amount of aldehydes in Ragusano cheese from milk from cows grazing native pasture (no details provided) compared to those fed concentrate (rye grass hay, corn meal, corn maize silage, cereal grains, oil seed by products, sugar by-products, minerals, methionine, butylated hydroxytoluene and etossichin). They found some aldehydes (phenylacetaldehyde, (*E*,*E*)-2,4-cotadienal, (*E*)-2-nonenal, (*Z*)-2-nonenal and 2,4-decadienal) were present only in the cheese produced from milk derived from pasture; and suggested that all apart from phenylacetaldehyde are the result of lipid oxidation of unsaturated FA in plants which have been transferred into the milk and then into cheese. Phenylacetaldehyde is involved in plant metabolism (synthesis of lignin) and was present in different forages and therefore even though also a product of amino acid metabolism during cheese ripening it also very possible that it was also transferred directly from pasture. Bovine milk from pasture was found to have elevated levels of 2-nonenal, hexanal, 2-heptanone, 1-pentanol, 2-nonanone and octanal than milk produced from concentrate (haylage, maize silage, grass hay, rolled barley grain, cracked corn grain, soybean meal, distillers grain and mineral mix) [20]. All of these compounds are potentially also derived from lipid oxidation in plants or milk fat. Bugaud et al. [54] produced Abondance cheese from milk from cows feed on pasture (gramineae, apiaceae and geraniaceae) in valley or mountain regions and cows fed only hay. Cheeses produced from mountainous regions were richer in aldehydes which again may be linked to lipid oxidation of plants or milk fat. Boltar et al. [69] noted that octanal and nonanal were significantly higher in winter Nanos cheese and were only present in grass silage and not in pasture, highlighting a possible direct transfer from forage to milk. Bovolenta et al. [70] found that some straight chain aldehydes such as acetaldehyde, hexanal, nonanal and benzaldehyde levels were significantly higher in nutrient poor pasture (poaceae, cyperaceae, asteraceae fabaceae and rosaceae) cheeses but some branched chain aldehydes (2-methylbutanal and 3-methylbutanal) were significantly higher in nutrient rich pasture (poaceae, cyperaceae, ranunculaceae and fabaceae) cheeses. Both 2-methylbutanal and 3-methylbutanal are common aldehydes produced from branched chain amino acid metabolism during cheese ripening. Stefanon and Procida [75] produced Montasio cheese from cows on 3 distinct diets; lucerne hay, lucerne hay and maize silage and from lucerne hay, maize silage and grass silage. There was virtually no dietary effect on twelve aldehydes identified in these cheeses.

In summary, it appears that a significant number of aldehydes can be transferred from plant material into milk and subsequently into cheese. Primary aldehydes are likely mainly derived from oxidation of PUFA from bovine milk fat and especially so in cheese over ripening. As aldehydes are very odour active they are likely to have a sensory influence, although this may be mitigated in cheese by the fact they are transitory compounds as they are readily metabolized.

### 5.2. Ketones

Stefanon and Procida [75] produced Montasio cheese from cows on 3 diets and noted significant effects were noted for diet and diet x ripening for most ketones. Levels of 2-pentanone, 3-methyl-2-pentanone or 6-methyl-5-hepten-2-one were all found to be influenced by diet. Glover et al. [20] noted that feeding concentrate raised levels of 2-pentanone and 2-decanone in milk in comparison to pasture feeding. Boltar et al. [69] noted a correlation between levels of some secondary alcohols and their corresponding methyl ketones, for example 2-heptanol and 2-heptanone were higher in Nanos cheese produced from winter milk and 2-nonanal and 2-nonanone were higher in Nanos cheeses produced in summer milk. Bendall and Olney [76] highlighted hept-*cis*-4-enal ((*Z*)-4-heptenal)) as an important volatile flavour component in milk derived from oxidation of LNA and thus is likely influenced by diet. Coppa et al. [57] found that another ketone, 2,3-octanedione was higher in milk derived from diverse pasture and suggested this was due to oxidation of LA and LNA which were higher in these milks. Boltar et al. [69] also found that 2-butanone content was higher in winter bovine milk produced from grass silage than in summer milk from grazing and again highlighted a possible direct transfer between forage and milk as 2-butanone is a common volatile produced in silage from carbohydrate metabolism. Mounchili et al. [77] indicated that 2-butanone was also a possible indicator of feed taints from baled silage (mixed grass legume). Toso et al. [67] found that acetone, 2,3-butanedione and 2-butanone best discriminated milk from different diets (lucerne hay, lucerne hay and maize silage, maize and grass silage). Verdier-Metz et al. [48] found that St-Nectaire type cheeses produced from bovine milk where cows were fed grass (natural native grass) silage contained higher levels of 3-hydroxy-2-butanone than the same cheeses produced from milk derived from hay (native grass). This may indicate that the carbohydrate content of the feed may be also impacting on the volatiles in milk as 3-hydroxy-2-butanone is primarily a product of carbohydrate metabolism. Carpino et al. [63] found 3-hydroxy-2-butanone in Ragusano cheese from milk from cows grazing native pasture (no details provided) but not in milk from cows fed concentrate (rye grass hay, corn meal, corn maize silage, cereal grains, oil seed by products, sugar by-products, minerals, methionine, butylated hydroxytoluene and etossichin). It may be that in this case 3-hydroxy-2-butanone may have also arisen from aspartic acid metabolism as the protein content of herbage based diets presents a higher protein to carbohydrate ratio [57].

Again, evidence exists for the direct transfer of ketones into milk, although this maybe a bigger issue with adverse sensory characteristics, especially with regard to a correlation with 2-butanone and conserved diets. Overall some studies have identified other ketones with specific diets but no obvious trends appear to exist. It also appears that ketones may not have a significant impact on milk flavour and most generated in cheeses are derived from oxidation of FA. Kalač [40] highlighted the fact that it is extremely difficult to ascertain the source of many aldehydes and ketones as some likely originate directly from the forage, some are produced during drying (hay) or in the preparation of silage or formed during cheese ripening. The transient nature of aldehydes may make them less important in cheese but their odour activity is higher than ketones and alcohols. Some important volatiles related to lipid oxidation are listed in Table 1.

### 5.3. Alcohols 

Ethanol is commonly found in silages and especially in maize silage and is thought to be directly transferred from forage to milk [71]. Toso et al. [67] identified volatiles in milk from cows on different diets (lucerne hay, lucerne hay and maize silage, maize and grass silage), and found that ethanol was a major discriminator based on diet. It is possible that alcohols have a more significant role in milk and cheeses derived from cows on preserved forages. Stefanon and Procida [75] produced Montasio cheese from cows on three distinct diets (hay-based; hay and maize silage; hay, maize silage and grass silage), alcohols were the most abundant volatile class and seven were significantly affected by diet (ethanol, 2-propanol, 1-propanol, 2-methyl-1-propanol, 1-penten-3-ol, 2-pentanol and 2-methyl-1-butanol). Bugaud et al. [54] produced Abondance cheese from milk of cows fed on pasture (gramineae, apiaceae and geraniaceae) in valley or mountain regions and cows fed hay only. Cheeses produced from cows fed hay contained more 2-methyl-butanol and 3-methyl-butanol. The source of all these alcohols is amino acid metabolism but again it may be due in some cases from direct transfer from the forage (possibly more likely from silage than hay, due to their high volatility, or from fermentation in the cheese over ripening). Some of the major alcohols potentially derived from diet in milk and cheese are listed in Table 1.

### 5.4. Acids

Short chain carboxylic acids are known to be important contributors to milk and especially cheese flavour. These acids are derived from lipolysis, carbohydrate metabolism or amino acid metabolism. Overall acids are often the most abundant volatile class in milk and cheese and some associations with levels in cheese have been linked to diet. Due to their volatility and concentration it could be assumed many carboxylic acids may be transferred directly from forage to milk. De Noni and Battelli [64] and Falchero et al. [78] found that FA content in artisanal cheeses reflected that of FA in milk derived from different alpine pastures. Villeneuve et al. [28] found that milk from cows fed (timothy) hay was characterized by higher contents of free FA than milk from cows on pasture (timothy). Bugaud et al. [54] produced Abondance cheese milk from cows fed on pasture (gramineae, apiaceae and geraniaceae) in valley or mountain regions and cows fed hay only. They found significant differences in volatile acids (acetic, butyric, propionic and caproic) and branched chain acids (2-methyl propionic acid and 3-methyl butanoic acids) between cheeses based on production region and diet. Caproic acid levels were highest in cheeses produced from valley milk and acetic and propionic acids were higher in cheeses derived from hay feeding. Similarly Carpino et al. [63] found acetic acid, butanoic acid were present at high concentrations in Ragusano cheese produced from pasture, or from a ration containing maize silage, rye-grass hay and concentrates (rye grass hay, corn meal, corn maize silage, cereal grains, oil seed by products, sugar by-products, minerals, methionine, butylated hydroxytoluene and etossichin). Bovolenta et al. [70] found that acids were the most prominent chemical class in Montasio cheeses but the only significant differences in acids between the cheeses produced from nutrient rich (poaceae, cyperaceae, ranunculaceae and fabaceae) and poor (poaceae, cyperaceae, asteraceae fabaceae and rosaceae) pasture were nonanoic and decanoic acids which were higher in cheeses produced from milk derived from nutrient rich pasture. Boltar et al. [69] found various free FA in Nanos cheese produced from winter (grass silage) and summer (grazing) milk throughout ripening but noted that pentanoic acid was only present in summer milk. These authors also found butanoic and hexanoic acids were contributing most to the total concentration of acids in Nanos cheese and that hexanoic acid was absent in summer milk. These authors highlighted a potential direct transfer between forage and milk for acetic acid, butanoic acid, 3-methyl butanoic acid and hexanoic acid. However, Carpino et al. [63] proposed that little if any acetic acid is directly transferred from diet to milk. Kalač [40] stated that pentanoic acid, hexanoic acid, 4-methylpentanoic acid (isocaproic acid) are indicators of poor silage and thus also suggests a potential direct transfer of these acids from silage diets.

Cornu et al. [44] investigated the impact of bovine milk from pasture (no details provided) and concentrate (flattened barley and soybean meal) in raw and pasteurized milk on the volatile profile of Cantal type cheese. Only 2-methylbutanoic acid, 3-methylbutanoic acid acids were significantly higher in concentrate fed cow’s milk cheese. The branched chain acids are derived from branched chain amino acid metabolism and this may be because more digestible protein exists in pasture than in conserved diets [57], however both these acids are also formed during cheese ripening. Stefanon and Procida [76] produced Montasio cheese from cows on three distinct diets; lucerne hay, lucerne hay and maize silage and from lucerne hay, maize silage and grass silage. They found that diet composition did not affect the volatile FA (acetic, propionic, butanoic and caproic) content in Montasio cheese.

These studies highlight the abundance of acids in milk and cheese, however conflicting results exist in relation to differences due to diet. FA content in cheese is dependent upon cheese type and age as well as FA content of the milk. However, evidence of direct transfer of short chain acids is apparent in milk either by ingestion or by inhalation. Some potentially important dietary related volatile acids are listed in Table 1.

### 5.5. Volatile Sulphur Compounds

As mentioned the high protein/non-fibrous carbohydrate ratio typical of grass diets enhances protein deamination by rumen microbes [57]. Dimethyl sulfone is derived by oxidation from dimethyl sulphide, which is metabolized from methionine and possibly cysteine in the rumen [28]. Bovolenta et al. [70] found that dimethyl sulfone was at highest levels in cheeses made from nutrient rich pasture (poaceae, cyperaceae, ranunculaceae and fabaceae). Dimethyl sulfone was also found to be at greater concentrations in cows fed pasture in comparison to hay with concentrate (maize, barley flakes and soybean cake) or silage (timothy) [28,57]. Coppa et al. [57] also found lower amounts of dimethyl sulfone in milk from hay and concentrates (maize, barley flakes and soybean cake) than from diverse pasture derived bovine milks. Faulkner et al. [41] also found that dimethyl sulfone was statistically higher in bovine milk from pasture (rye-grass) and pasture (rye-grass) plus white clover than in milk derived from concentrates (grass silage, maize silage, maize, beet pulp, soybean meal, salt, rapeseed meal and megalac). Toso et al. [67] identified sulphur volatiles in milk from cows on different diets; lucerne hay, lucerne hay and maize silage, maize and grass silage and found that dimethyl sulfone was a major discriminator based on diet. Carpino et al. [63] found dimethyl sulphide and methionol also derived from methionine in Ragusano cheese from milk from cows grazing diverse native pasture but not in milk from cows fed concentrate (rye grass hay, corn meal, maize silage, cereal grains, oil seed by products, sugar by-products, minerals, methionine, butylated hydroxytoluene and etossichin). Mounchili et al. [77] indicated that dimethyl sulphide was a possible indicator of feed taints from baled silage (mixed grass legume). In contrast Stefanon and Procida [75] produced Montasio cheese from cows fed on three distinct diets; lucerne hay, lucerne hay and maize silage and from lucerne hay, maize silage and grass silage and found that only methyl disulphide was influenced by diet. Bugaud et al. [54] produced Abondance cheese from milk from cows fed on pasture (gramineae, apiaceae and geraniaceae) in valley or mountain regions and cows fed hay only and found that cheese produced from valley regions had the least sulphur compounds.

Sulphur volatiles are potentially very important aroma compounds due to their high odour activity and intense aroma [78]. Dimethyl sulfone in cheese has been linked to pasture diets in a number of studies [28,41,57,67,70] and therefore is an important pasture derived volatile and a potential biomarker for pasture derived dairy products. It is also likely to influence sensory perception (Table 1) due to its low odour threshold.

### 5.6. Esters

There are two main enzymatic mechanisms for the bio-synthesis of esters; esterification and alcoholysis. Esterification is the formation of esters from alcohols and carboxylic acids, whereas alcoholysis is the production of esters from alcohols and acylglycerols or from alcohols and fatty acyl-CoAs derived from the metabolism of FA, amino acids and/or carbohydrates [26]. Therefore, ester content is dependent upon the content of primary alcohols and carboxylic acids. Kalač [40] suggested carboxylic acids originating from plants or produced during ensiling and alcohols formed in silage produce various esters. Coppa et al. [57] found higher levels of esters in diverse pasture derived milk, suggesting that the source was likely enzymatic esterification of short chain alcohols and free FA after milking. Bovolenta et al. [70] found that ester concentration was high in cheeses but only isopropyl butanoate was significantly higher in cheeses made from nutrient rich pasture (poaceae, cyperaceae, ranunculaceae and fabaceae). Carpino et al. [63] found a significant amount of esters in Ragusano cheese from milk derived from cows grazing native pasture with those fed concentrate (rye grass hay, corn meal, maize silage, cereal grains, oil seed by products, sugar by-products, minerals, methionine, butylated hydroxytoluene and etossichin). The following esters were only present in the cheese produced from cows fed pasture; ethyl-2-methyl butyrate, geranyl acetate, (*E*)-methyl jasmonate. The esters geranyl acetate and (*E*)-methyl jasmonate are also likely derived directly from pasture as both are also products of lipid oxidation in plants when plants are damaged. Toso et al. [67] identified volatiles in milk from cows on different diets, lucerne hay, lucerne hay and maize silage, maize and grass silage. They found two esters best discriminated the samples; ethyl acetate and ethyl isovalerate (ethyl-3-methyl butanoate). Boltar et al. [69] highlighted a potential direct transfer between forage and milk for ethyl hexanoate, ethyl octanoate and ethyl decanoate. These authors also found that ethyl hexanoate was higher in winter bovine milk produced from grass silage than in summer milk from grazing and found the opposite effect for ethyl acetate which correlated with the forage. They also noted that most esters identified in Nanos cheese were ethyl esters, with ethyl butanoate the most prominent acid in both summer and winter Nanos cheese. Stefanon and Procida [75] produced Montasio cheese from cows on three distinct diets (lucerne hay, lucerne hay and maize silage and from lucerne hay, maize silage and grass silage). Seventeen esters were identified and 6 were found to be significantly affected by diet; ethyl propionate, ethyl hexanoate, butyl acetate, propyl acetate, propyl propionate and propyl hexanoate. The presence of ethyl esters is linked to the availability of ethanol which maybe higher in milks and cheese derived from conserved forage especially silage as ethanol has been previously associated with silage [71] as mentioned earlier. The most commonly associated esters linked to diet in milk and cheese is listed in Table 1.

### 5.7. Lactones

Lactones are cyclic compounds formed by the intramolecular esterification of hydroxyacids through the loss of water [79]. Lactones in general are described as having a buttery-type character and can be derived by heat in the presence of water and hydroxyacids, with γ- and δ-lactones derived from 4- and 5-hydroxy fatty acids, respectively. Thus, lactones are more likely in heat-treated milk than in raw milk. Villeneuve et al. [28] found that sweet flavour was higher in milk from cows fed hay and suggested a potential link to levels of γ-lactones in the milk. Bendall [67] evaluated the volatile profile of two milks from separate diets; pasture (rye-grass, white clover and small amounts of other grasses and weeds) or from cows fed a supplement diet (maize silage, grass silage, hay, cottonseed, maize grain, barley, soybean, fishmeal, vegetable oil, fat, corn gluten, molasses, minerals and vitamins) that were identified as having distinct flavours. However, only one volatile γ-12:2 lactone was absent from one milk sample and present in the other out of a total of 71 aroma active volatile compounds. Villeneuve et al. [28] noted that milk from cows fed hay (timothy) was characterized by higher contents of lactones than milk from cows on pasture (timothy). Bovolenta et al. [70] found that γ-butyrolactone was higher in cheeses made from poor pasture than nutrient rich pasture (poaceae, cyperaceae, ranunculaceae and fabeceae).

In general, few differences in lactone content were linked to diet but it is likely they are more important in pasteurized milk and pasteurized milk cheese than in raw milk or raw milk cheese because heat is a factor in their production. Some potentially dietary lactones are listed in Table 1.

### 5.8. Toluene

Recent studies have highlighted toluene as a potential biomarker for pasture derived milk and cheese [52,65] as it is a product of β-carotene degradation in the rumen [28]. Levels correspond with levels of β-carotene in pasture, which are highest in fresh forage [3,57]. Boltar et al. [69] found that toluene was present in higher levels in summer milk. Croissant et al. [3], Villeneuve et al. [28] and Faulkner et al. [41] also found toluene at highest concentrations in milk derived from a pasture based system and Cornu et al. [44], Bovolenta et al. [70] and Boltar et al. [69] found significantly higher levels in cheeses made from pasture. Boltar et al. [69] also highlighted a potential direct transfer of toluene from feed to milk and subsequently into Nanos cheese. However, O’Callaghan et al. [52] noted that toluene levels were also influenced by ripening time in Cheddar cheese. Stefanon and Procida [75] produced Montasio cheese from cows on three distinct diets; lucerne hay, lucerne hay and maize silage and from lucerne hay, maize silage and grass silage and found that toluene was not affected by diet. However, this probably highlights the fact that carotenoid content may be low in these hay and silage diets due to losses. Aprea et al. [80] did not find any significant difference in toluene levels in Montasio cheese from cows’ milk derived from pasture and pasture plus supplements (commercial mixed concentrate), which is not surprising as the β-carotene levels would be similar in both diets. Toluene has been described with a wide range of sensory attributes; nutty, sweet, almond [52], sweet, pungent, caramel, ethereal, fruity/rubbery [69], plastic [44] and was linked to rancidity by [57]. Faulkner et al. [41] did not find an obvious direct link between toluene and the sensory character of milks derived from pasture (rye-grass), pasture (rye-grass) seeded with white clover and concentrate (grass silage, maize silage, maize, beet pulp, soybean meal, salt, rapeseed meal, megalac). This is not surprising as toluene has a high odour threshold in comparison to other volatiles.

In summary toluene levels are higher in pasture derived milk as it is product of β-carotene metabolism in the rumen and is subsequently transferred to the milk. Toluene may also be a biomarker for pasture derived dairy products and more research is required to ascertain specific levels in dairy products. As its odour threshold is high it is very unlikely to an impact on the sensory characteristics of milk or cheese.

### 5.9. Phenolic Compound

Phenolic compounds may also be important volatile compounds related to forage intake in milk and cheese. Alkylphenols in ruminant milks are derived from phenolic compounds ingested through feed [56]. Forages are also rich in cellular phenolic compounds [4]. Conjugated alkylphenols in milk constitute a reservoir for species-related alkylphenols in dairy products. Species related *p*- and *m*-cresols and 3- and 4-ethylphenols were found to be mostly conjugated with sulphate with minor amounts associated with phosphate and glucuronide conjugates in all milks [81]. Alkylphenols, such as 3- and 4-ethylphenols are thought to be important in ovine milk flavour, with other alkylphenols such as 2-ethylphenol, m-cresol and phenol important flavour compounds in other ruminant milks. Kilic and Lindsay [81] found mostly sulphate conjugates of *p*- and *m*-cresols and 3- and 4-ethylphenols in bovine, caprine and ovine milks. Lopez and Lindsay [82] found that *p*-cresol was the major alkylphenol in bovine milk and has a barn-yard like odour, with m-cresol having a medicinal odour [82]. Alkylphenols exist mainly as sulphate conjugates in ruminant milks when phenolic compounds or their precursors are ingested via feed. Lopez and Lindsay [82] noted that phenolic compounds were responsible for the cowy flavour of cow’s milk. Faulkner et al. [41] found that *p*-cresol was likely responsible for an increase in barnyard aroma of bovine milks from rye-grass and rye-grass seeded with white clover over those produced by concentrate (grass silage, maize silage, maize, beet pulp, soybean meal, salt, rapeseed meal, megalac). These authors also suggested the increase in *p*-cresol was also likely due to increased degradation of tryptophan and tyrosine due to more digestible protein in the pasture diets as well as the potential degradation of an isoflavone, possibly formonenetin which is known to be present in leguminous plants such as clover [81]. Cow odour was linked to *p*-cresol in Montasio cheese made from nutrient poor pasture. The level of *p*-cresol in the same cheeses produced with pasture and a low level of supplementation (commercial mixed concentrate) were similar to those on nutrient poor pasture yet these could not be perceived as different for cow odour, suggesting that other volatiles may mask this effect or be involved [80]. Khanal et al. [51] found that milk derived from pasture (no details provided) feeding had a more pronounced barny flavour than milk from cows fed concentrate (alfalfa hay, corn silage, seam-flaked corn, grain, cottonseed, soybean meal, sugar beet pulp, yeast mix, molasses and fat) only but analysis was not undertaken to determine if *p*-cresol was higher in the pasture derived milk. Bovolenta et al. [70] found significant differences were evident in phenolic (phenol, *p*-cresol, *m*-cresol and 4-allyl-phenol) compounds between cheeses derived from different bovine diets, with *p*-cresol and 4-allyl-phenol higher in cheeses from nutrient poor (poaceae, cyperaceae, asteraceae fabaceae and rosaceae) pasture and phenol and m-cresol higher in nutrient rich (poaceae, cyperaceae, ranunculaceae and fabaceae) pasture cheeses. Moio et al. [55] has linked *p*-cresol with the degradation of lignin in plants and thus it may also be transferred directly from forage into milk. Carpino et al. [63] found vanillin in Ragusano cheese from milk from cows grazing native pasture (no details provided) but not in milk from cows fed a concentrate (flattened barley and soybean meal) diet. Vanillin is a phenolic aldehyde and the authors also suggested a link to lignin synthesis in plants and thus this compound may be also derived directly from pasture. Other potential phenolic compounds that may influence flavour—such as skatole and indole—are produced by the deamination and decarboxylation of tryptophan in the rumen [57] or in ensiled forage. Croissant et al. [3] and Coppa et al. [57] found higher levels of skatole and indole in pasture derived milk than milk from concentrate diets.

Studies appear to indicate an impact of phenolic compounds related to diet on the sensory character of milk and cheese, with *p*-cresol likely responsible for barny or cow flavours in dairy products. The main source of *p*-cresol in milk and cheese also appears to be rumen metabolism of β-carotene and aromatic acids.

## 6. Sensory Perception of Raw Bovine Milk

Most sensory methods applied to milk can be summarized into three main types; descriptive, discrimination (usually triangle tests) or affective (consumer or ranking) tests.

In relation to raw milk, Guichard et al. [83] found significant sensory differences in raw milk derived from a range of different pastures (pasture with different grasses, pasture with little diversity including undesirable plants, diverse pasture with aromatic plants, diverse pasture with legumes) using a trained descriptive panel (Table 2). These differences included, overall odour intensity, overall aroma intensity, animal flavour, hay odour, butter odour, butter aroma, cream odour, white colour, cream odour, fresh milk odour and sugar flavour. Dubroeucq et al. [84] also found sensory differences in raw milks based on diet using discriminative sensory analysis (triangle tests). They found that untrained panellists (consumers) could discern that raw milk derived from pasture was different from that derived from meadow hay (*p* < 0.001), or from concentrate (barley and soybean) (*p* < 0.001). They also discriminated raw milk derived from grass (rye-grass) silage from meadow hay (*p* < 0.01) and maize silage (*p* < 0.05) but could not discern any differences in raw milk derived from meadow hay, maize silage, concentrate (barley and soybean), or meadow hay enriched with aromatic plants (Table 2). These studies clearly identify a sensory impact of raw milk based on diet. They also imply that the sensory impact of different diets (pasture and different types of conserved forage) can be perceived by both trained and untrained panellists (consumers) and that the effect varies according to the type of diet and possibly other on-farm factors. These studies indicate that milk from pasture may be most different from milk derived from conserved forage and concentrate diets. Verdier-Metz et al. [85] evaluated raw milk by discriminate analysis (triangle tests) from hay native mountain pasture and rye-grass silage. They found that 45% of assessors correctly identified milk source (*p* < 0.05) and milk derived from rye-grass silage was deemed stronger flavour.

Tornambé et al. [86] evaluated raw bovine milk produced from maize silage with different levels of added essential oil (0.1 and 1.0 μL of oil/L of milk) distilled from mountain pasture using discriminative analysis (triangle tests). Untrained assessors did not find significant sensory differences at low addition levels (0.1 μL/L) but did at high addition (1.0 μL/L) levels (*p* < 0.001) (Table 2), highlighting a dietary component concentration factor influencing sensory perception. Other studies on raw milk have not found any differences in sensory perception using trained descriptive sensory panels or discriminative sensory analysis with trained or untrained panellists. Lerch et al. [87] using a trained descriptive panel did not find any significant difference in raw milk derived from maize silage and from maize silage with added linseed oil (or added linseed oil and vitamin E) in terms of odour (intensity and cream), basic taste (sugar), in the mouth perception for taste or odour (intensity, cream and watery) or texture in the mouth (thick and pellicle) (Table 2). These same authors also undertook discriminate (triangle tests) analysis with a trained panel on the same milks but also could not discern any sensory difference (Table 2). Again, this study highlights potential concentration factors, where the concentration of the added oils was either insufficient to affect sensory perception or that any potential impact was lessened by other factors during digestion or transfer to the mammary gland. The same applies to a study by Bovolenta et al. [88] who used discriminative sensory analysis (triangle tests) with trained panellists. Raw milk from cows grazing mountain pasture supplemented with two levels (4.8 and 1.6 kg per day) of concentrate (maize, sugar beet pulp, wheat bran, brewer’s yeast, dry alfalfa, barley, soybean, sunflower meal, minerals and vitamins) could not be distinguished (Table 2). In another study, Tomambé et al. [7], also using discriminate testing (triangle tests) with untrained panellists, could not discern any sensory differences in raw milk derived from cows on temporary grassland with 17 grass species, from raw milk derived from cows on permanent grassland with 31 or 50 grass species (Table 2). It is possible that as the diets were all pasture based, sensory differences if present were too subtle to be perceived by the untrained panel.

An adverse impact of feeding silage on the sensory character of raw milk was highlighted in studies by Mouchilli et al. [77] and Bertilssion and Murphy [89]. Mouchilli et al. [77] investigated changes in the sensory quality of raw milk using discriminative analysis using trained panellists (Table 2). They found that raw milk from cows fed barley concentrate was always deemed good quality; however, 78% of milk 30 min post feeding timothy and clover silage were deemed inferior and had developed an off-flavour. Bertilssion and Murphy [89] fed cows three different types of silage (rye-grass, red clover and white clover) and found that the percentage of raw milk deemed acceptable (normal) by trained panellists (discriminative sensory analysis) was lower for red clover silage and white clover silage (58% and 64%, respectively) than rye-grass silage (85%) (Table 2).

In summary, it is difficult to discern specific sensory effects from different types of pasture or conserved diets on raw milk due to differences in farm factors and the diversity of the sensory approaches used. However, it is evident that diet can influence the sensory properties of raw bovine milk in certain circumstances. It also appears that it is easier to discern raw milk derived from pasture from all other diets (conserved forage and concentrates). Addition of supplements can also have a sensory impact that appears unsurprisingly to be concentration dependent. Some studies have also highlighted a potential adverse impact of silage especially incorporating clover on sensory perception.

## 7. Sensory Perception of Pasteurized Bovine Milk

Fresh pasteurized bovine milk has a delicate bland flavour, which is characterized by sweet aromatic, cooked and milk fat notes [3]. It is well established that heat-treatment can alter the volatile profile of milk and thus its sensory perception [61]. Obviously the greater the intensity of heat-treatment the greater the potential sensory impact [61]. The most common heat-treatment(s) for milk for general consumption is high temperature short time pasteurization or ultra-high temperature (UHT) processing. Heat-treatment can promote the formation of numerous volatile compounds in milk that have the potential to alter sensory perception, for example; the decarboxylation of α-keto acids to generate methyl ketones or lactones [43,61,90], the oxidation of methanethiol to sulphur compounds [91], the degradation of β-carotene [92], or the generation of Maillard reaction products [93].

Croissant et al. [3] undertook a detailed sensory study of pasteurized milk from cows fed pasture (mixture of grasses) supplemented with maize (corn) and cottonseed, in comparison to cows fed concentrate (maize silage, alfalfa haylage, grain concentrate, whole cottonseed, soybean hulls, pelleted corn gluten, vitamins and minerals). Using a trained descriptive panel significant sensory differences were found. Milk from cows fed concentrate was associated with a sweeter feed/malty flavour, a greater sweet aromatic flavour and a sweet taste (*p* < 0.05), with milk derived from pasture plus supplements having a distinct salty taste (*p* < 0.05) and grassy and mothball flavours (*p* < 0.05) (Table 3). These milks were also evaluated using discrimination testing (triangle tests) using untrained panellists, however no significant sensory differences (overall liking, flavour liking or texture/mouthfeel) were found (Table 3). An affective (75 untrained consumers) study was also undertaken with no significant differences in overall consumer acceptance noted. This study highlights that untrained panellists (consumers) maybe less likely to discern sensory differences than trained panellists in pasteurized milk, it also highlights a subtle impact of diet on milk in relation to flavour. A study by Khanal et al. [51] also found differences between trained and untrained panellists. This group conducted descriptive and affective sensory tests on pasteurized milk produced from cows fed pasture (details not provided), pasture plus concentrate (grain and minerals) or concentrate (alfalfa hay, corn silage, seam-flaked corn, grain, cottonseed, soybean meal, sugar beet pulp, yeast mix, molasses and fat). Trained panels found greater barny flavours (*p* < 0.05) in milks derived from pasture and pasture plus concentrate than milk derived from cows fed only concentrate (Table 3). However, regular consumers using an affective sensory ranking test did not discern any differences for mouthfeel, colour, flavour or overall milk quality (Table 3).

Villeneuve et al. [28] used an affective consumer sensory ranking test to compare pasteurized milk derived from pasture (timothy) and hay (timothy) and pasteurized milk derived from hay and silage (timothy). They found that pasteurized milk derived from pasture had a significantly greater total flavour (raw milk, fresh milk and farm milk) and grassy flavour (grass, leafy vegetable and plant) (*p* < 0.05) and that pasteurized milk from hay had a greater sweet flavour (empyreumatic, vanilla, caramel and sugar) (*p* < 0.05) (Table 3). This group also undertook discrimination (triangle tests) testing and found that untrained panellists could discern differences between pasteurized milk derived from pasture from hay (*p* < 0.01) but not milk derived from silage from hay (Table 3). Thus, in this study both trained and untrained panellists (consumers) could distinguish sensory differences in pasteurized milk derived from pasture and hay but were unable to discern differences in pasteurized milk derived from silage from hay.

Moorby et al. [45] using a trained descriptive panel found that pasteurized milk was significantly whiter and viscous (*p* < 0.001) derived from grass silage (rye-grass) than from red clover silage and that pasteurized milk derived from red clover had a significantly higher boiled milk flavour (*p* < 0.001) but no other sensory (creamy aroma, intensity of milk odour, buttery aroma, oily aroma, cream flavour, sweet flavour, sour flavour, plastic flavour, metallic flavour, mouthfeel, aftertaste or overall liking) differences were detected (Table 3). Faulkner et al. [41] using an untrained panel and a ranked descriptive sensory test found a greater intensity of barny aroma (*p* < 0.05) in pasteurized milk derived from pasture (rye-grass) and pasture (rye-grass) seeded with white clover than milk derived from concentrate (grass silage, maize silage, maize, beet pulp, soybean meal, salt, rapeseed meal, megalac) (Table 3). These authors also found that milk derived from pasture was more viscous and less white (*p* < 0.05) than milk derived from concentrate. The impact of diet on pasteurized milk colour and viscosity is evident in these studies [26,28] which could be discerned by both trained and untrained panellists.

Other studies have found an adverse impact on the sensory properties of pasteurized milk when maize was present in concentrate or in silage fed to cows. Mogensen et al. [94] using a trained descriptive sensory panel found that increasing the portion of maize in concentrate (grass/clover silage, maize silage, oats, field beans) fed to cows subsequently increased maize odour and sour feed attributes (*p* < 0.05) in the resultant pasteurized milk. These authors also found that added toasted field beans to the concentrate did not influence sensory attributes (Table 3). Larsen, et al. [9] also using a trained descriptive panel found that as the portion of maize in maize:lucerne silage increased, stale aroma, stale flavour and creamy flavour (*p* < 0.05) increased in the pasteurized milk derived from these silages (Table 3).

Lejonklev et al. [95] investigated the impact of adding different levels of oregano or caraway oils (0.2 and 1.0 g of oil/kg of dry matter of feed) to concentrate (barley, soybean, rapeseed, grass/clover silage, maize silage and minerals) on the sensory characteristics of the resultant milks with different levels of added oregano and caraway oil. They found that a trained panel could not distinguish milk derived from concentrate and milk derived from concentrate with added caraway oil but could detect a significant increase in stored corn aroma (*p* < 0.05) and a decrease in fresh aroma (*p* < 0.05) in milk derived from concentrate with high levels of added oregano oil (Table 3). Again, this highlights an obvious concentrate impact of the additive but in this case, it was perceived in pasteurized milk.

Other studies using descriptive sensory analysis and trained panels did not find any significant sensory differences in milk due to diet. Shingfield et al. [96] did not find any differences in pasteurized milk derived from grass (timothy and meadow fescue) silages with or without additives (formic acid, orthophosphoric acid and Enzymax) and hay (timothy and meadow fescue) using a trained descriptive panel (Table 3). Gaillard et al. [97] evaluated the impact of different protein concentrations (14% and 16%) in feed using different sources of protein (soybean meal canola caked and died distiller’s grains with solubles) on pasteurized milk quality. Using descriptive sensory analysis with a trained panel they found no significant sensory difference (Table 3). Finally Yayota et al. [98] using an affective sensory ranking test with untrained panellists evaluated pasteurized milk produced from cows fed either grass (reed canarygrass) silage, maize silage, or soybeans/brewer grain but did not find any sensory (sweetness, body, texture, aftertaste and palatability) differences (Table 3).

Overall it appears that the impact of different forage types on the sensory properties of pasteurized bovine milk is complex with some distinct effects or no effects. This obviously depends upon individual fed type and other on farm factors. An apparent impact of carotenoids level in milk correlating with yellow colour intensity was evident in some studies, with a good correlation between pasture feeding and pasteurized milk colour. A number of studies have also linked pasture diets to a barney sensory attribute in pasteurized bovine milk. A potential correlation of diet and sensory viscosity of milk was also highlighted which is likely linked to the fatty acid content of milks. Other studies have noted a potential negative impact of maize or legumes (clover) in silage or concentrate on the sensory properties of pasteurized milk. Certain additives also appear to have a sensory effect, which is both additive and concentration dependent. It is noteworthy that untrained assessors which better represent consumers often seem to be less able to discriminate sensory differences in pasteurized milk based on diet than trained assessors. This highlights that when differences exist they are often likely to be quite subtle and that visual or sensory rheological differences are more likely to be perceived than flavour aspects.

## 8. Sensory Perception of Raw and Pasteurized Bovine Milk Cheese

Quite a number of studies investigating the impact of diet on the sensory character of cheese have been undertaken both on raw and pasteurized milk cheeses. The consequences of milk pasteurization on cheese characteristics is well documented, apart from eliminating pathogenic bacteria it also modifies the biochemistry and microbiology of cheese ripening, influencing the flavour and texture of the cheese [44].

### 8.1. Soft and Semi-Soft Cheeses 

Both Bovolenta et al. [70] and Aprea et al. [80] analysed the impact of milk from nutrient rich and poor pasture varying in vegetation types on the sensory characteristics of Montasio cheese (a raw milk bovine soft cheese). Bovolenta et al. [70] used descriptive sensory analysis (10 trained assessors evaluated 26 attributes) and found that cheeses ripened for 60 days produced from milk derived from cows on a nutrient rich pasture (poaceae, cyperaceae, ranunculaceae and fabaceae) had a significantly higher colour intensity (*p* < 0.04) and a higher granular texture (*p* < 0.03) than those on nutrient poor pasture (poaceae, cyperaceae, asteraceae fabaceae and rosaceae). These authors found that cow odour (*p* < 0.04), cow flavour (*p* < 0.01), adhesive texture (*p* < 0.03) and creaminess texture (*p* < 0.03) were significantly higher in cheeses made from milk of cows fed the nutrient poor pasture (Table 4). These same authors did not find any sensory difference in cheeses from milk of cows fed both nutrient rich and poor pasture with different levels of concentrate (maize, barley, beet pulp, soy and wheat). Aprea et al. [80] also evaluated the impact of nutrient rich and nutrient poor pasture (similar feeds as [71]) with high or low supplements (commercial mixed concentrate) on the sensory properties of Montasio cheese ripened for 12 months using descriptive sensory analysis (12 trained assessors evaluating 28 attributes). They found that 10 attributes were significantly influenced by diet; colour intensity (*p* < 0.01), sour odour (*p* < 0.05), humid appearance (*p* < 0.01), pungent odour (*p* < 0.01), bitterness (*p* < 0.05) and granular texture (*p* < 0.01) which were significantly more pronounced in cheeses produced from a nutrient rich pasture. Cheeses from a nutrient poor pasture had a greater eye distribution (*p* < 0.05), more rough appearance (*p* < 0.01) and a cow odour (*p* < 0.05) (Table 4). Cheeses from nutrient rich and poor pasture supplemented with concentrate were not significantly different [71]. This implies that the addition of supplement may remove differences due to nutrient rich and poor pasture in relation to sensory perception of the resultant milk. Romanzin et al. [50] undertook a consumer study using 280 untrained assessors on Montasio cheese produced from mountain pasture plus concentrate (maize, barley, beet pulp, soybean and wheat) or from alfalfa hay, meadow hay plus concentrate (maize, barley, beet pulp, soybean and wheat) at 60 days and 180 days of ripening. Montasio cheese produced from mountain pasture plus concentrate was deemed too dark in colour (*p* < 0.01) with too few holes (*p* < 0.05 and *p* < 0.01) at 60 and 180 days in comparison to the cheese produced from alfalfa hay, meadow hay and concentrate had a better colour (*p* < 0.01, *p* < 0.05) and a better number of holes (*p* < 0.01) (Table 5).

Verdier-Metz et al. [48] evaluated the sensory quality of St-Nectaire type cheeses. St Nectaire cheese is an uncooked, pressed semi-soft cheese made from raw or pasteurized bovine milk. The authors produced the cheeses from raw bovine milk derived from cows fed grass silage (natural grassland) and from cows fed hay (natural grassland) which was ripened for 8 weeks. The only significant sensory differences relating to diet from descriptive sensory (10 trained assessors evaluated 15 attributes) analysis were that cheeses produced from the milk of cows fed grass silage were significantly more yellow (*p* < 0.01) and that opening number (*p* < 0.01) was higher than cheeses produced from milk derived from grass hay (Table 4). Verdier-Metz et al. [49] also produced St-Nectaire type cheese from cows fed 3 distinct hay diets (cocksfoot, rye-grass and native mountain grassland), ripened for 7 weeks They also undertook descriptive evaluation (10 trained assessors for 18 attributes) but the only significant difference was that cheeses produced from milk derived from cocksfoot hay and concentrate had more regular openings (*p* < 0.05) than the other cheeses (Table 4). These same authors also undertook aroma analysis using 11 untrained assessors evaluating 13 aroma attributes. They found only 1 significant odour difference with cheeses produced from native mountain grassland hay and concentrate having a greater cabbage odour (*p* < 0.05) (Table 6). They also undertook consumer testing (using 8 trained assessors) and evaluated 3 attributes (appearance, texture and taste) and found that cheeses produced from native mountain grassland hay scored significantly higher for appearance (*p* < 0.01) and taste (*p* < 0.01) and that cheeses produced from rye-grass hay and concentrate scored significantly higher for texture (*p* < 0.01) and taste (*p* < 0.01). Thus, cheeses from cocksfoot hay scored lowest for each attribute (Table 5). Verdier-Metz et al. [85] undertook further studies on raw milk St-Nectaire cheese ripened for 7 weeks which was produced from cows milk on differing diets of rye-grass silage (with added formic acid) plus concentrate (soybean meal and barley) and mountain grassland hay plus concentrate, (soybean meal and barley). Using descriptive sensory analysis (10 trained assessors and 33 attributes) the only significant difference was that cheeses made from milk of cows fed rye-grass silage had a more gritty texture (*p* < 0.05) (Table 4). Overall very little differences were found between grass silage and grass hay diets on the sensory character of St-Nectaire cheese by Verdier-Metz et al. [48,49,85] despite undertaking descriptive, discriminate and consumer sensory studies. It is possible that the addition of concentrate may have minimized sensory differences from preserved forages.

These studies on soft and semi-soft cheeses have highlighted that diet is often seen to influence changes in colour and appearance and that cheeses produced from milk from cows on nutrient poor pasture is often associated with cow flavours. Also, the addition of concentrate with forage appears to offset sensory differences from nutrient rich and poor pasture in the resultant cheeses. Some studies have also linked cocksfoot hay diets with lower sensory scores that native mountain or rye-grass hay.

### 8.2. Semi-Hard Cheeses

A study on Cantal cheese (at 6, 13 and 23 weeks) evaluated the sensory impact of bulk milk collected from two extreme groups of farms differing in intensity of production in summer, autumn, winter and spring [42]. Cantal cheese is made from raw or pasteurized bovine milk. In this study, the first group had a low intensification of dairy cow management; small herds that mainly calved in winter that were fed almost exclusively pasture during the grazing period (minimal concentrate supplementation (no details provided) and with hay during winter. The second group had higher stocking rates, higher producing herds, calving mainly throughout the year and foraged temporary meadows supplemented with preserved forage and winter rations of wrapped haylage supplemented with concentrates (no details provided). Cantal cheese was made from raw milk and descriptive sensory analysis (12 trained assessors evaluating 69 attributes) found that the cheese-making period and ripening time had a much greater impact on the overall sensory character of the cheeses than the intensification of milk production. However, the authors also noted that cheese-making period (time of year) was also linked to differences in the diet. Cheeses produced from the second group produced cheeses that were more salty (*p* < 0.05), bitter (*p* < 0.05), pungent (*p* < 0.05), with a more intense odour (*p* < 0.001) and butter aroma (*p* < 0.001) than those from the first group. Cheeses made from the first group were more elastic (*p* < 0.001) (Table 4). The effect of production differed depending upon period of production and ripening time and the effect was less noticeable in summer and autumn than in winter or spring. This study highlights ripening time appears to be more important that diet in relation to sensory aspects and that production time of year is also more important than diet. However, some clear sensory differences were noted between the different production systems that were at least in part dietary related. In another study on raw milk Cantal cheeses ripened for 3 months produced from cows on two distinct diets; a rye-grass silage diet and a mountain grassland hay diet, both included concentrates (soybean meal and barley) [85]. Descriptive sensory analysis (10 trained assessors used 43 attributes) found that cheeses produced from mountain grassland hay had significantly better curd appreciation (*p* < 0.01), curd flavour (*p* < 0.01), melting texture (*p* < 0.001) and mellow texture (*p* < 0.01), with more butter colour (*p* < 0.05) and grass odour (*p* < 0.05), citrus-fruit aroma (*p* > 0.05) and persistent flavour (*p* > 0.05) than cheeses produced cows on a rye-grass silage diet. Cheeses from the rye-grass silage were characterized as having an increased alcohol odour (*p* < 0.001) and chemical aroma (*p* < 0.05) (Table 4). Tornambé et al. [86] also produced raw Cantal cheese from maize silage with different levels of added essential oil distilled from mountain pasture (dose rates provided in Section 6). These authors found Cantal cheese produced from milk ripened for 5 months derived from maize silage with a high dose of essential oil had a significantly altered descriptive sensory profile (12 trained assessors using 38 attributes) than cheeses produced with low levels of essential oil or no added essential oil (Table 4). Cornu et al. [44] investigated the impact of raw and pasteurized bovine milk from pasture (no details provided) with concentrate (flattened barley and soybean meal) and from hay with concentrate (flattened barley and soybean meal) on the sensory properties of Cantal type cheese ripened for 6 months, using 18 untrained assessors evaluating 9 aroma attributes. A number of significant impacts of diet were noted (Table 6). Hazelnut aroma (*p* < 0.03) was higher in milk derived from hay and concentrate in 3-month-old cheeses. Raw milk cheeses at 3 months, had a greater intense aroma (*p* < 0.002), rancid aroma (*p* < 0.005), musty aroma (*p* < 0.0001) and pungent aroma (*p* < 0.001), where pasteurized cheeses at 3 months had a greater cream aroma (*p* < 0.003) and hazelnut aroma (*p* < 0.02). At 6 months cheeses from pasture plus concentrate had a greater musty aroma (*p* < 0.01) and cheeses from hay plus concentrate had a more butter aroma (*p* < 0.02), cream aroma (*p* < 0.02) and hazelnut aroma (*p* < 0.02). Raw milk cheeses at 6 months had a more intense aroma (*p* < 0.008), rennet aroma (*p* < 0.03) and musty aroma (*p* < 0.03), with pasteurized milk cheeses having a greater cream aroma (*p* < 0.009). This study highlights a significant impact of pasteurization as well as diet.

Buchin et al. [71] produced Abondance cheese from raw milk derived from mountain pastures of different diversity with concentrate (rolled cereal mixture). Abondance cheese is a semi-hard raw milk cheese. Descriptive sensory analysis was undertaken (13 trained assessors using 58 attributes) on 5-month-old cheese. They found significant sensory differences in cheeses produced from different mountain pastures on north and south facing slopes in summer (Table 4) with differences in both texture and taste. Bugaud et al. [54] also produced Abondance cheese from raw milk of cows feed on pasture in valley or mountain regions or from cows fed only hay. Descriptive sensory analysis was undertaken (14 trained assessors who evaluated 9 attributes) and they found that Abondance cheese ripened for 24 weeks produced from milk of cows fed on mountain alpine pasture had significantly higher levels of fruity aroma (*p* < 0.05), hazelnut aroma (*p* < 0.01), boiled milk odour (*p* < 0.01), fresh cream odour (*p* < 0.05), bread crust aroma (*p* < 0.05) and animal aroma (*p* < 0.001). Cheeses produced with hay had a greater fruity aroma (*p* < 0.05) and pungent taste (*p* < 0.01), with cheeses produced from valley pasture having a greater bread-crust aroma (*p* < 0.05), pungent taste (*p* < 0.001) and propionic acid aroma (*p* < 0.05) (Table 4). This study also investigated cheeses produced within each diet and also found significant sensory variation, highlighting the fact that differences of on farm factors and production factors also influence the sensory properties of cheese.

Khanal et al. [51] investigated the sensory quality of Cheddar cheese produced from pasteurized milk from grazing cows, grazing cows supplemented with a grain mix (soybeans, corn, beet pulp and molasses) and from cows fed concentrate (alfalfa hay, corn silage, steam-flaked corn, grain mix, whole linted cottonseed, soybean meal, sugar beet pulp and molasses). Cheddar cheese is a semi-hard pasteurized milk cheese produced in English speaking countries. They carried out quantitative descriptive analysis (7 trained assessors evaluating 16 attributes) and found no significant difference in sensory perception of the Cheddar cheeses ripened for 40 days (Table 4). These authors also undertook a consumer study with 83 untrained assessors and evaluated 4 attributes (colour, mouth-feel, flavour and overall quality) and also did not find any significant sensory (Table 5) difference. O’Callaghan et al. [52] evaluated the effect of pasture (rye-grass), pasture (rye-grass) seeded with white clover and milk from cows indoor feeding on concentrate (grass silage, maize silage, maize, beet pulp, soybean meal, salt, rapeseed meal, megalac) using ranked descriptive analysis (25 untrained assessors familiar with Cheddar cheese were asked to rank the cheeses for 20 attributes) on pasteurized Cheddar cheese ripened at 180 and 270 days. The authors found that at 180 d of ripening, cheeses made from milk from cows fed rye-grass pasture were significantly positively associated with colour and negatively with sweet taste, while cheeses made from milk where cows were fed rye-grass pasture seeded with white clover were negatively associated with crumbly texture and cheeses made from milk where cows were fed concentrate were significantly negatively associated with colour and positively with fruity/estery flavour (Table 4). In relation to the twenty attributes evaluated only four were found to differ significantly at 180 days ripening. At 270 days of ripening cheeses produced from milk where cows were fed rye-grass pasture with or without seeded white clover were significantly positively associated with colour, cheeses produced from milk of cows fed rye-grass pasture were also positively associated with salt taste. Cheeses produced from milk of cows fed rye-grass pasture were also negatively associated with sweet taste and cheeses from milk of cows fed concentrate were negatively associated with colour but positively associated with sour taste. Again, only four out of twenty attributes evaluated were significantly different at 270 days ripening. O’Callaghan et al. [52] also undertook a consumer study and found that at 180 d ripening cheeses produced from cows fed with rye-grass pasture seeded with white clover had significantly higher scores for liking of appearance and overall acceptability. Cheeses produced from cows on concentrate scored significantly higher for liking of texture and overall acceptability at 180 days of ripening. The only significant difference at 270 days of ripening was that cheeses produced from cows fed rye-grass pasture scored significantly lower for liking of appearance and had a lower score for liking of texture (Table 5).

These studies on semi-hard cheese produced contrary results in relation to the impact of diet on the sensory perception of cheese. Some evidence suggests that the impact of pasteurization is greater than any dietary impact. Other studies highlighted a significant effect of diet on appearance (colour), texture and flavour. One study also highlighted significant variance within replicate cheese trials produced from milk derived from the same diets, which highlights that cheese microflora and cheese production parameters may be more influential than diet.

### 8.3. Hard Cheeses

Carpino et al. [99] produced Ragusano cheese from raw bovine milk where cows were fed two distinct forages; diverse native pasture supplemented with concentrate (rye grass hay, corn meal, maize silage, cereal grains, oil seed by products, sugar by-products, minerals, methionine, butylated hydroxytoluene and etossichin) or solely concentrate (rye grass hay, corn meal, corn maize silage, cereal grains, oil seed by products, sugar by-products, minerals, methionine, butylated hydroxytoluene and and etossichin). Ragusano cheese is a raw milk pasta filata cheese. Descriptive sensory analysis (11 trained assessors scored for 26 attributes) was undertaken using 4-month-old cheese. Cheeses produced from milk of cows fed pasture supplemented with concentrate were significantly more yellow (*p* < 0.05), had a greater floral odour (*p* < 0.05) and a green/herbaceous odour (*p* < 0.05) than those cheeses produced from cows fed only concentrate (Table 4). Out of the twenty-six descriptive attributes only four were found to be statistically different. Cheeses produced from concentrate had significantly greater fracture consistency (*p* < 0.05) and more of an oily consistency (*p* < 0.05) than cheeses produced from milk of cows on pasture and concentrate. Bovolenta et al. [88] produced Nos-trano di Primiero cheese from the raw milk of cows grazing mountain pasture with added high and low levels of concentrate (maize, sugar beet pulp, wheat bran, brewer’s yeast, dry alfalfa, barley, soybean, sunflower meal, minerals and vitamins). Nos-trano di Primiero cheese is an alpine washed rind raw milk hard cheese. These authors carried out descriptive sensory analysis (8 trained assessors evaluating 35 attributes) and found that cheeses ripened for 60 d produced from milk of grazing cows with a high supplement were classified as been significantly more bitter (*p* < 0.05), more soluble (*p* < 0.05), firmer (*p* < 0.05) and more granular (*p* < 0.05) (Table 4). While cheeses produced from cows with a low supplement had a significantly more adhesive texture (*p* < 0.05). These authors also undertook difference testing (13 trained assessors) using triangle tests but did not find any significant sensory differences (Table 5).

Only two studies have been carried out on the impact of diet on hard cheeses and both studies included concentrate which may have mitigated the impact of pasture diets on the sensory perception of these cheeses based on previous studies. However, only a minority of the descriptive sensory attributes assessed in both cases were found to differ and most of these were related to colour or texture and not flavour.

### 8.4. Stretched Curd Cheeses

Bonanno et al. [100] produced Caciovallo Palermitano raw milk cheese from different cow breeds (Cinisara and Brown) on separate diets. Caciovallo Palermitano is a raw milk cheese. The Cinisara cows were feed pasture (no details provided), hay with concentrate (no details provided) and the Brown cows fed hay some concentrate (no details provided) and a little pasture (no details provided). They undertook a full descriptive analysis (12 trained assessors evaluating 14 attributes) on 1 month old cheese. The cheese produced from Cinisara cows fed pasture, hay and concentrate had a significantly smoother texture (*p* < 0.001), a sweeter taste (*p* < 0.01) and a more acidic taste (*p* < 0.001). The cheese from Brown cows fed hay, concentrate and a little pasture, was characterized by significantly more bitterness (*p* < 0.001) and saltiness (*p* < 0.05). Esposito et al. [101] compared a raw bovine milk Caciocavallo cheese produced from a pasture based feeding (natural species plus seeded rye-grass and clover) system to a confined (grain and hay) system. Descriptive sensory analysis was undertaken (12 trained assessors evaluating 17 attributes) and the authors found that cheeses produced from pasture were more yellow (*p* < 0.0001), had a greater spicy taste (*p* < 0.002) and seasoned taste (*p* < 0.003) and a greater friability (*p* < 0.0001) and a greater grainy texture (*p* < 0.002) (Table 4). Cheese produced from grain and hay had a significantly more uniform appearance (*p* < 0.0001), a greater butter odour/flavour (*p* < 0.05) and smoked odour/flavour (*p* < 0.002) and a bitter taste (*p* < 0.003). These authors also used a consumer acceptability study (100 untrained assessors scored for overall liking, appearance, taste/flavour and texture) but did not find any significant sensory difference between the cheeses (Table 5).

Both of these studies on stretched curd cheeses found sensory differences due to diet and as these cheeses were evaluated when relatively young this may be a significant factor as the biochemistry of cheese ripening is less advanced.

## 9. Conclusions

A distinct impact of pasture diet on the sensory properties of milk and cheese is increased yellow intensity, which is correlated to β-carotene content. Overall it appears the impact of different forage types on the sensory properties of milk and cheese is very complex. Evidence supports the direct transfer of many volatiles from grazing or from conserved forage, some of which are related to plant (forage) metabolism or fermentation during conservation processes but many volatiles are also transferred indirectly post rumen metabolism. Some studies have shown a direct impact of pasteurization on the volatile profile of milk, with formation of new volatiles and reduction or elimination of others. The impact of volatiles from diet on sensory perception of milk and cheese depends upon their concentration and odour activity although little quantitative studies have been carried out. Studies have also shown that the impact of pasteurization of milk can be more significant than diet on volatile profiles in cheese. In addition, the length of cheese ripening may mitigate sensory differences due to diet as more volatile aromatic and non-volatile flavour compounds increase due to the metabolic and enzymatic activities of bacteria, yeasts and moulds. However, other studies have tentatively associated some specific volatiles with diet; for example, dimethyl sulfone with pasture diets (due to an increase in available protein) and ethanol with conserved forages (direct transfer) but this is difficult to verify due to the range of on farm and processing factors that may also be involved. Stronger cases exist for the association of β-carotene, toluene and *p*-cresol in milk and cheese with pasture diets. A definitive sensory link has not been made with toluene possibly due to its high aroma threshold but it appears its main pathway into milk is from the rumen digestion of β-carotene. The sensory attributes barny and cowy aroma/flavour appear to be linked to *p*-cresol content in milk and cheese, which is also appears to be derived from the metabolism of β-carotene but also from the metabolism of aromatic amino acids and possibly isoflavones in the rumen. Terpenes may also influence the sensory properties of milk and dairy products if derived from wild natural pastures where they are very abundant and concentrations are much higher than in managed monocultures (dicotyledones typically contain much more terpenes than monocotyledons). The odour activity of terpenes is low and these compounds can be both biosynthesized and bio-converted in the rumen and during cheese ripening making it difficult to link them as specific biomarkers for pasture derived milk and cheese, apart from maybe milk and cheese derived from wild mountain pastures. The development of off-flavours in milk and cheese has also been associated with silages that contain maize or legumes (mainly clover). Diet influences the FA profile of milk with higher levels of PUFA in milk derived from pasture, increasing levels of omega-3 FA. However even though some products of lipid oxidation may be higher in milk and cheese from pasture, it appears that natural anti-oxidants elevate adverse impacts on the sensory characteristics in milk and the natural reducing environment of cheese seems to mitigate any effect but that is less likely to be the case in butter and whole milk powders. Some studies have highlighted that increased β-carotene levels in pasture derived milk may also reduce susceptibility to photo-oxidation. Changes in fat content and FA profile appear to influence the textural perception of milk and dairy products. Studies have confirmed that the ratio of oleic acid to palmitic acid impacts the texture of milk, butter and cheese. It also appears that conserving forage may also reduce the sensory impact derived from the use of fresh forage in cheese. Many studies have highlighted that pasture diets appear to have the greatest impact on milk flavour, as these tend to be more easily discerned by trained panellists and consumers. However, other studies have highlighted that the addition of concentrate with pasture may reduce dietary sensory effects in milk and cheese. In relation to the sensory evaluation of milk and cheese it is noted that untrained assessors which best represent consumers appear less able to discriminate sensory differences in milk and cheese than trained assessors and that differences in visual (mainly colour) and textural attributes were easier to discern that flavour attributes. This likely suggests that sensory differences due to diet are often subtle.

## Figures and Tables

**Table 1:** Potentially important volatiles derived from diet in bovine milk and cheese.

Volatiles	Chemical Class	Potential Factor(s)	Odour Description
Hexanal	Aldehyde	Lipid Oxidation (Oleic and Linoleic acid)	Cardboard like, metallic off flavour, green
Propanal	Aldehyde	Lipid Oxidation (Linolenic and Docosahexaenoic acid)	Solvent-like, acrid
Pentanal	Aldehyde	Lipid Oxidation (Arachidonic and linoleic acid)	Cardboard like, metallic off flavour, green
(*Z*)-4-Heptenal	Aldehyde	Lipid Oxidation (Linolenic acid)	Oily, fatty, green, milky, creamy, dairy
Benzaldehyde	Aldehyde	Amino acid metabolism (Phenylalanine)	Bitter almond, sweet cherry
Phenylacetaldehyde	Aldehyde	Lignin metabolism, amino acid metabolism (Phenylalanine)	Honey-like, rosy, violet-like, hyacinth, green
2-Methyl propanal	Aldehyde	Amino acid metabolism (Leucine)	Banana, malty, chocolate-like, cocoa
2-Methyl butanal	Aldehyde	Amino acid metabolism (Isoleucine)	Malty, dark chocolate, almond, cocoa, coffee
3-Methyl butanal	Aldehyde	Amino acid metabolism (Leucine)	Malty, cheese, green, dark chocolate, cocoa
Ethanol	Alcohol	Carbohydrate metabolism, plant diet	Dry alcohol
1-Hexanol	Alcohol	Lipid Oxidation (hexanal)	Green, floral
1-Pentanol	Alcohol	Lipid Oxidation (pentanal)	Fruity, alcoholic, green, balsamic, woody
2-Methyl propanol	Alcohol	Amino acid metabolism (2-Methyl propionic acid)	Alcohol, wine-like, plastic
2-Methyl butanol	Alcohol	Amino acid metabolism (2-Methyl butanoic acid)	Malty
3-Methyl butanol	Alcohol	Amino acid metabolism (3-Methyl butanoic acid)	Fresh cheese, breath-taking, alcoholic, fruity, solvent-like, floral, malty
3-Octen-2-one	Ketone	Lipid Oxidation (Arachidonic and Linoleic acid)	Mushroom-like
3,5-Octadien-2-one	Ketone	Lipid Oxidation (Arachidonic and Linoleic acid)	Mushroom-like
2,3-Octanedione	Ketone	Lipid Oxidation (Linoleic and Linolenic acid)	Dill, cooked broccoli, buttery
1-Octen-3-one	Ketone	Lipid Oxidation (Linoleic and 1-Octen-3-ol)	Metallic, mushroom-like
3-hydroxy-2-butanone (acetoin)	Ketone	Carbohydrate metabolism, Amino acid metabolism (Aspartic acid)	Buttery, sour milk, caramel
2-Butanone	Ketone	Carbohydrate metabolism, plant diet	Buttery, sour milk
Acetic acid	Acid	Carbohydrate metabolism, amino acid metabolism (Aspartic acid or methionine), plant diet	Vinegar, peppers, green, fruity floral, sour
Butanoic acid	Acid	De-novo synthesis and lipolysis	Sweaty, butter, cheese, strong, acid, faecal, rancid, dirty sock
Hexanoic (Caproic acid)	Acid	De novo synthesis and lipolysis	Sweaty, cheesy, sharp, goaty, bad breath,
2-Methyl butanoic acid	Acid	Amino acid metabolism (2-Methyl butanal)	Fruity, waxy, sweaty-fatty acid
2-Methyl propionic acid	Acid	Amino acid metabolism (2-Methyl propanal)	Fruity
3-Methyl butanoic acid	Acid	Amino acid metabolism (2-Methyl butanal)	Cheesy, sweaty, rancid, faecal, rotten fruit, goat
4-Methylpentanoic acid (isocaproic acid)	Acid	Plant diet, amino acid metabolism (leucine)	Pungent, cheesy
Limonene	Terpene	Plant diet	Citrus
β-pinene	Terpene	Plant diet	Herbaceous
α-Pinene	Terpene	Plant diet	Pine, green
*p*-Cymene	Terpene	Plant diet	Citrus, woody, spicy
δ-3-Carene	Terpene	Plant diet	Sweet, citrus
Sabinene	Terpene	Plant diet	Woody, citrus, pine, spicy
Camphene	Terpene	Plant diet	Woody
β-Caryophyllene	Sesquiterpene	Plant diet	Sweet, woody, spicy, clove, dry
β-Phellandrene	Sesquiterpene	Plant diet	Mint, peppery, slightly citrus
α-Phellandrene	Sesquiterpene	Plant diet	Terpenic
γ-Cadinene	Sesquiterpene	Plant diet	Herbal, woody
Geranyl acetate	Ester	Oxidation of plant diet and oxidation of dimethyl sulphide	Sweet, fruity, citrus
Ethyl hexanoate (ethyl caproate)	Ester	Ethanol and hexanoic acid, plant diet	Fruity, malty, young cheese, mouldy
Ethyl butanoate	Ester	Ethanol and butanoic acid, plant diet	Buttery, ripe fruit, sweet
Dimethyl disulphide	Sulphur	Photo oxidation, plant diet, amino acid metabolism (Methionine)	Cabbage-like, garlic, green, sour, onion
Dimethyl sulfone	Sulphur	Oxidation of dimethyl sulphide, plant diet	Sulphurous, hot milk, burnt
Toluene	Hydrocarbon	Carotenoid metabolism (β-carotene), plant diet	Nutty, bitter, almond, plastic (high odour threshold)
*p*-Cresol	Phenol	Carotenoid metabolism (β-carotene, isoflavone), amino acid metabolism (Tryptophan and Tyrosine), plant diet	Barny, cowy, medicinal
γ-12:2 lactone	Lactone	Lipid oxidation (Linolenic acid), heat-treatment	Muesli with honey, baby powder
γ-Butyrolactone	Lactone	Lipid oxidation, heat treatment	Creamy, oily, fatty nuances

**Table 2:** Summary of sensory analysis studies of raw bovine milk.

Sensory Test Details	Diets	Significant Difference	Reference
Descriptive			
23 Attributes 15 Trained assessors	Primarily monoculture of ryegrass (G1)	(G1) Sugar flavour (*p* < 0.05)	[83]
	Pasture will little diversity but with mainly grasses (G2)	(G2) Animal odour (*p* < 0.01)	
		(G2) Hay odour (*p* < 0.05)	
		(G2) Animal flavour (*p* < 0.05)	
	Pasture will little diversity but with undesirable plants (G3)	(G3) Overall odour intensity (*p* < 0.05)	
		(G3) Butter odour (*p* < 0.05)	
		(G3) Cream odour (*p* < 0.05)	
		(G3) White colour (*p* < 0.05)	
		(G3) Butter aroma (*p* < 0.05)	
	Diverse pasture with many aromatic plants (G4)	(G4) White colour (*p* < 0.05)	
		(G4) Sugar flavour (*p* < 0.05)	
	Diverse pasture with lots of legumes (G5)	(G5) Cream odour (*p* < 0.01)	
		(G5) Animal odour (*p* < 0.01)	
		(G5) Animal flavour (*p* < 0.01)	
		(G5) Overall aroma intensity (*p* < 0.05)	
		(G5) Fresh milk odour (*p* < 0.05)	
		(G5) Hay odour (*p* < 0.05)	
Temperature 22 °C 19 Attributes 10 Trained assessors	Maize silage	No significant differences (*p* < 0.10)	[87]
	Maize silage + linseed oil		
	Maize silage + linseed oil + vitamin E		
**Discriminate Testing**	**Diets**	**Significant Difference**	**Reference**
Temperature 4 °C 12 Untrained assessors Triangle test	Pasture (natural pasture) Hay (meadow hay) Grass silage (ryegrass) Maize Silage Concentrate (high and low) (barley and soybean) Hay plus aromatic plants	Pasture vs. hay—(*p* < 0.001)	[84]
		Pasture vs. concentrate—(*p* < 0.001)	
		Grass silage vs. hay—(*p* < 0.01)	
		Grass silage vs. maize silage—(*p* < 0.05)	
		Hay vs. maize silage—Not different	
		Hay vs. concentrate—Not different	
		Hay vs. hay enriched with aromatic plants—not different	
Temperature 16 °C 16 Untrained assessors Triangle test	Maize Silage with added essential oil (distilled from mountain grassland pasture) at two levels	No significant difference at low dose of essential oil Significant difference at high dose of essential oil (*p* < 0.001)	[86]
Temperature chilled 2 Trained assessors	Silage Perennial Ryegrass Silage Red clover Silage White clover	15% of PR milk deviated from normal 42% of RC milk deviated from normal 36% of WC milk deviated from normal	[89]
Room temperature	Barley concentrate	All milk samples from cows feed barley concentrate were good quality	[77]
5 Trained assessors	Mixed grass (timothy) clover silage Mountain grassland Temporary grassland Permanent grassland Species rich permanent grassland	78% of milk samples taken from cows 30 min after consuming silage were deemed as having an off-flavour No significant difference	[7]
Milk heated to 45 °C Temperature 20 °C 10–11 Assessors Triangle test	Initially all cows on maize silage plus concentrate Ryegrass silage (formic acid + concentrate, mins and vitamins as required) Mountain grassland hay (concentrate, minerals and vitamins as required)	45% correctly identified milk from cows feed Ryegrass silage and Mountain grassland hay (*p* < 0.05) 23% identified based on texture and 51% based on taste Milk from Ryegrass silage was stronger	[85]
Temperature 16 °C 17 Trained assessors Triangle Test	Mountain pasture with supplements Pasture + Low level of Concentrates (maize, sugar beet pulp, wheat bran, brewer’s yeast, dried alfalfa, barley, soybean flakes, sunflower meal, minerals and Vitamins) low level Pasture + High level of Concentrates (as above)	No significant difference	[88]

**Table 3:** Summary of sensory analysis studies of pasteurized bovine milk.

Sensory Test Details	Diets	Significant Difference	Reference
Descriptive			
Temperature 22 °C 20 Attributes 8 Trained assessors	Pasture (alfalfa) Pasture (alfalfa) + grain mix with soybeans, beet pulp, corn and molasses or soybean, beet, corn and molasses Concentrate (alfalfa hay, maize silage, corn, grain mix, lilted cottonseed, soymeal, sugar beet pulp, yeast mix, molasses and fat)	Pasture: Barny (*p* < 0.05) Pasture (alfalfa) + grain mix: Barny (*p* < 0.05) No significant differences in overall quality or colour between diets	[51]
Temperature 15 °C 4 Attributes 6 Assessors	Hay (timothy + meadow fescue) Silage (timothy + meadow fescue) Silage plus enzymes (timothy + meadow fescue) Silage plus formic acid mix (timothy + meadow fescue)	No significant differences	[96]
Temperature 15 °C 10 Attributes 10 Trained assessors	Pasture supplemented with corn and cottonseed Concentrate (maize silage, alfalfa haylage, grain concentrate, whole cottonseed, soybean hulls, pelleted corn gluten, vitamins and minerals)	Pasture + Supplement, Mothball, Grassy, Salty (*p* < 0.05) Concentrate; Sweet (*p* < 0.05) aromatic, sweet, sweet, feed, malty (*p* < 0.05)	[3]
Temperature 15 Attributes	Grass silage (ryegrass) Red clover silage at different ratio’s 66% Grass, 33% Red clover silage 33% Grass, 66% Red clover silage	Grass Silage; Whiteness (*p* < 0.01) Texture more viscous and creamier (*p* < 0.001) Red Clover Silage increased boiled milk flavour (*p* < 0.01), No significant differences in preference (aroma, aftertaste or overall liking)	[45]
Temperature 16 °C 14 Attributes 8 Trained assessors	Concentrate (oats, grass clover silage) + two (high and low) levels of maize silage + treated or untreated field beans	Concentrate with high maize silage + toasted field beans: Intense sour feed odour (*p* < 0.05) Concentrate + high maize silage + untreated field beans: Intense maize odour (*p* < 0.05) No effect of toasting on overall sensory quality Maize % in the concentrate positively correlated with maize odour	[94]
Temperature 7 °C 12 Attributes 30 Untrained assessors	Pasture (timothy) Hay (timothy) Silage (timothy)	Pasture Total flavour (*p* < 0.05) Grassy flavour (*p* < 0.05) Hay Sweet flavour (*p* < 0.05)	[28]
Temperature 16–18 °C 15 Attributes 10 Trained assessors	Maize:Lucerne silage (5:1) Maize:Lucerne silage (2:1)	Maize:Lucerne silage (5:1) Higher stale aroma (*p* < 0.05) Higher creamy flavour (*p* < 0.05) Higher stale flavour (*p* < 0.05)	[9]
Temperature 7–10 °C 5 Attributes 23 Untrained assessors	Concentrate (hay, beet pulp, alfalfa hay and formula) with Grass silage (canary grass) Concentrate (as above) with maize silage Concentrate (as above) with by product (soybean curd or brewers grain)	No significant differences (sweetness, body, texture, aftertaste or palatability)	[98]
Temperature 16–18 °C 15 Attributes 8–10 Trained assessors	Concentrate (barley, soybean, rapeseed, grass/clover silage, maize silage and minerals) Concentrate (as above) + high or low doses of caraway oil Concentrate (as above) + high or low doses of oregano oil	Concentrate with high dose of oregano oil: Lower fresh aroma (*p* < 0.05), Higher corn aroma (*p* < 0.05), Higher stored aroma (*p* < 0.05)	[95]
Temperature 21 °C 18 Attributes 25 Untrained Assessors	Pasture (ryegrass) Pasture (ryegrass) + white clover Concentrate (grass silage, maize silage, maize, beet pulp, soybean meal, salt, rapeseed meal, megalac)	Pasture: Colour (*p* < 0.05), Increased viscosity (*p* < 0.05) Pasture + white clover: Colour (*p* < 0.05) and Barnyard aroma (*p* < 0.05)	[41]
Temperature 4 °C 15 Attributes 9 Trained Assessors	A high and low protein mix with soybean-canola, beet pulp, A high and low protein mix with dried distillers grain with soluble	No significant differences	[97]
**Discriminate Testing**	**Diets**	**Significant Difference**	**Reference**
Temperature 4 °C 50 Untrained assessors Triangle test	Pasture supplemented with corn and cottonseed Concentrate (maize silage, alfalfa haylage, grain concentrate, whole cottonseed, soybean hulls, pelleted corn gluten, vitamins and minerals	No significant difference	[3]
Temperature 7 °C Triangle test 30 Untrained assessors	Pasture (timothy) Hay (timothy) Silage (timothy)	Silage vs. Hay—No significant difference Pasture vs. Hay—(*p* < 0.01)	[28]
**Consumer Testing**	**Diets**	**Significant Difference**	**Reference**
Temperature 22 °C Consumer acceptability 62–92 Untrained assessors	Pasture (alfalfa) Pasture (alfalfa) + grain mix with soybeans, beet pulp, corn and molasses or soybean, beet, corn and molasses Concentrate (alfalfa hay, maize silage, corn, grain mix, lilted cottonseed, soymeal, sugar beet pulp, yeast mix, molasses and fat)	No significant difference	[51]
Temperature 4 °C 75 Untrained assessors Hedonic Assessment	Pasture supplemented with corn and cottonseed Concentrate (maize silage, alfalfa haylage, grain concentrate, whole cottonseed, soybean hulls, pelleted corn gluten, vitamins and minerals)	No significant difference	[3]

**Table 4:** Summary of descriptive sensory analysis studies bovine cheese.

Cheese and Age	Analysis	Diet	Result	Reference
Raw milk Cantal-Type 6, 13 and 23 weeks	12 Trained assessors 69 Attributes	Pasture (minimal concentrate addition) and hay in winter	More elastic (*p* < 0.001)	[42]
		Pasture (temporary meadows), with preserved forage and haylage in winter with concentrates	More salty taste (*p* < 0.05) More bitter taste (*p* < 0.05) More pungent taste (*p* < 0.05) More odour intensity (*p* < 0.001) More butter odour (*p* < 0.001)	
Raw milk Cantal 3 months	10 Trained assessors 43 Attributes	Mountain grassland hay + concentrate (soybean meal and barley)	Curd Appreciation (*p* < 0.01) Curd Flavour (*p* < 0.01) Melting texture (*p* < 0.001) Mellow texture (*p* < 0.01) Butter colour (*p* < 0.05) Grass odour (*p* < 0.05) Citrus fruit aroma (*p* < 0.05) Persistent flavour (*p* < 0.05)	[85]
		Ryegrass silage (with formic acid) + concentrate (soybean meal and barley)	Alcohol aroma (*p* < 0.001) Chemical aroma (*p* < 0.05)	
Raw milk Cantal 5 months	12 Trained assessors 38 Attributes	Maize silage + low dose (0.1 μL/L) essential oil	Highest butter odour (*p* < 0.001) Highest fresh cream odour (*p* < 0.001) Highest cooked cheese odour (*p* < 0.001) Highest vanilla odour (*p* < 0.001) Lowest mint odour (*p* < 0.001) Highest lactic acid aroma (*p* < 0.001) Highest garlic aroma (*p* < 0.05) Highest stubble aroma (*p* < 0.01) Highest meat aroma (*p* < 0.001) Lowest thyme/oregano aroma (*p* < 0.001) Highest salt taste (*p* < 0.01) Lowest astringent taste (*p* < 0.001) Lowest persistent taste (*p* < 0.001)	[86]
		Maize silage + high dose (1.0 μL/L) essential oil	Highest odour intensity (*p* < 0.001) Lowest butter odour (*p* < 0.001) Lowest fresh cream odour (*p* < 0.001) Lowest acidified cream odour (*p* < 0.001) Lowest cooked cheese odour (*p* < 0.001) Lowest vanilla odour (*p* < 0.001) Lowest brioche odour (*p* < 0.001) Lowest meat odour (*p* < 0.01) Highest mint odour (*p* < 0.001) Highest aroma intensity (*p* < 0.001) Lowest butter aroma (*p* < 0.001) Lowest cooked cheese aroma (*p* < 0.001) Lowest lactic acid aroma (*p* < 0.001) Lowest garlic aroma (*p* < 0.05) Lowest grilled onion aroma (*p* < 0.001) Lowest stubble aroma (*p* < 0.01) Lowest meat aroma (*p* < 0.001) Lowest cheese mites’ aroma (*p* < 0.001) Highest mint/chlorophyll aroma (*p* < 0.001) Highest thyme/oregano aroma (*p* < 0.001) Lowest salt taste (*p* < 0.01) Highest astringent taste (*p* < 0.001) Highest persistent taste (*p* < 0.001)	
		Maize silage control	Highest acidified cream odour (*p* < 0001) Highest cooked cheese odour (*p* < 0.001) Highest brioche odour (*p* < 0.001) Highest meat odour (*p* < 0.01) Lowest mint odour (*p* < 0.001) Highest aroma intensity (*p* < 0.001) Highest butter aroma (*p* < 0.001) Highest cooked cheese aroma (*p* < 0.001) Highest garlic aroma (*p* < 0.05) Highest grilled onion aroma (*p* < 0.001) Highest cheese mites’ aroma (*p* < 0.001) Lowest mint/chlorophyll aroma (*p* < 0.001) Lowest thyme/oregano aroma (*p* < 0.001) Lowest persistent taste (*p* < 0.001)	
Raw Milk St-Nectaire 8 weeks	10 Trained assessors 15 attributes	Silage (mountain grass including cocksfoot) + concentrate (soybean meal and barley)	Curd colour (more yellow) (*p* < 0.01)	[48]
		Hay (mountain grass including cocksfoot) + concentrate (soybean meal and barley)	More openings (*p* < 0.01)	
Raw Milk St-Nectaire 8 weeks	10 Trained assessors 18 Attributes	Cocksfoot hay + concentrate (cereal and soybean)	More opening regularity (*p* < 0.05)	[49]
		Rye-grass rich hay + concentrate (cereal and soybean)	No Significant Differences	
		Native mountain grassland hay + concentrate (cereal and soybean)	No Significant Differences	
Raw Milk St-Nectaire 7 weeks	10 Trained assessors 33 Attributes	Ryegrass silage (with formic acid) + concentrate (soybean meal and barley)	Gritty texture (*p* < 0.05)	[85]
		Mountain grassland hay + concentrate (soybean meal and barley)	No Significant Differences	
Raw Milk Abondance 5 months	13 Trained assessors 58 Attributes	South facing pasture + concentrate (rolled cereal mix) (15–21 June)	Less persistent taste (*p* < 0.05) More firm texture (*p* < 0.001) More grainy microstructure (*p* < 0.01) More smooth microstructure (*p* < 0.001)	[71]
		North facing pasture + concentrate (rolled cereal mix) (22–29 June)	Less firm (*p* < 0.001) More adhesive texture (*p* < 0.05) Thinner microstructure (*p* < 0.01) Less grainy microstructure (*p* < 0.01) Higher salty taste (*p* < 0.001) More bitter taste (*p* < 0.01) More persistent taste (*p* < 0.05)	
		South facing pasture + concentrate (rolled cereal mix) (30 June–6 July)	More firm texture (*p* < 0.001) More grainy microstructure (*p* < 0.01) Less persistent taste (*p* < 0.05)	
Raw Milk Abondance 24 weeks	14 Trained assessors 9 Attributes	Mountain pasture	Higher fruity aroma (*p* < 0.05) Higher hazelnut aroma (*p* < 0.01) Higher boiled milk odour (*p* < 0.01) Higher fresh cream odour (*p* < 0.05) Higher bread crust aroma (*p* < 0.05) Higher animal aroma (*p* < 0.001)	[54]
		Hay	Higher fruity aroma (*p* < 0.05) Higher pungent taste (*p* < 0.01)	
		Valley pastures	Higher bread crust aroma (*p* < 0.05) Higher pungent taste (*p* < 0.001) Higher propionic acid aroma (*p* < 0.05)	
Pasteurized Milk Cheddar 40 days	Temperature 21 °C 7 Trained assessors 16 Attributes	Alfalfa hay + corn silage + concentrate (corn, grain, cottonseed, soybean meal, sugar beet pulp, yeast mix, molasses and fat)	No Significant Differences	[51]
		Pasture	No Significant Differences	
		Pasture + grain mix (soybeans, corn, sugar beet pulp and molasses)	No Significant Differences	
Pasteurized Milk Cheddar 180 and 270 days	Temperature 21 °C 25 Untrained assessors 20 Attributes	Pasture (rye-grass) at 180 days ripening	Highest colour (*p* < 0.002) Lowest sweet taste (*p* < 0.002)	[52]
		Pasture + white clover at 180 days ripening	Lowest crumbly texture (*p* < 0.026)	
		Concentrate (grass silage, maize silage, maize, beet pulp, soybean meal, salt, rapeseed meal and megalac) at 180 days ripening	Lowest colour (*p* < 0.001) Highest fruity estery flavour (*p* < 0.05)	
		Pasture (rye-grass) at 270 days ripening	Highest colour (*p* < 0.001) Highest salty taste (*p* < 0.001)	
		Pasture (rye-grass) + white clover at 270 days ripening	Lowest sweet taste (*p* < 0.013) Highest colour (*p* < 0.001)	
		Concentrate (grass silage, maize silage, maize, beet pulp, soybean meal, salt, rapeseed meal and megalac) at 270 days ripening	Lowest colour (*p* < 0.001) Highest sour taste (*p* < 0.007)	
Raw Milk Montasio 60 days	Temperature 21 °C 10 Trained assessors 26 Attributes	Nutrient rich pasture (poaceae, cyperaceae, ranunculaceae and fabaceae)	Higher colour intensity (*p* < 0.04) Higher granule texture (*p* < 0.03)	[70]
		Nutrient poor pasture (poaceae, cyperaceae, asteraceae fabaceae and rosaceae)	Higher cow odour (*p* < 0.04) Higher cow flavour (*p* < 0.01) Higher adhesive texture (*p* < 0.03) Higher creaminess texture (*p* < 0.03)	
		Nutrient rich and poor pasture + 3 kg/head of concentrate (maize, barley, beet pulp, soy, wheat)	No Significant Difference	
		Nutrient rich and poor pasture + 1.5 kg of concentrate (maize, barley, beet pulp, soy, wheat)	No Significant Difference	
Raw Milk Montasio 12 months	Temperature 21 °C 12 Trained assessors 28 Attributes	Nutrient rich pasture (poaceae, cyperaceae, ranunculaceae and fabaceae)	Higher colour intensity (*p* < 0.01) Greater sour odour (*p* < 0.05) More humid appearance (*p* < 0.01) Greater pungent odour (*p* < 0.01) Greater bitterness (*p* < 0.05) More granular texture (*p* < 0.01)	[80]
		Nutrient poor pasture (poaceae, cyperaceae, asteraceae fabaceae and rosaceae)	Greater eye distribution (*p* < 0.05) More rough appearance (*p* < 0.01) Greater cow odour (*p* < 0.05)	
		Nutrient rich and poor pasture + 3 kg/head of concentrate (maize, barley, beet pulp, soy, wheat)	No Significant Differences	
		Nutrient rich and poor pasture + 1.5 kg of concentrate (maize, barley, beet pulp, soy, wheat)	No Significant Difference	
Raw Milk Ragusano 4 months	Temperature 21 °C 11 Trained assessors 26 Attributes	Native pasture + concentrate (rye-grass hay, corn meal, corn silage, cereal grains, oil seeds, sugar by-products, minerals, etossichin)	More yellow colour (*p* < 0.05) More floral odour (*p* < 0.05) More green/herbaceous (*p* < 0.05)	[99]
		Concentrate (rye-grass hay, corn meal, corn silage, cereal grains, oil seeds, sugar by-products, minerals, etossichin)	More oily consistency (*p* < 0.05) More fracture consistency (*p* < 0.05)	
Raw Milk Nostrano di Primiero 60 days	Temperature 16 °C 8 Trained assessors 35 Attributes	Pasture + low level of concentrates (maize, sugar beet pulp, wheat bran, brewer’s yeast, dried alfalfa, barley, soybean flakes, sunflower meal, minerals and vitamins) 1.6 kg dry matter/day	More irregularity shaped eyes (*p* < 0.05) More adhesiveness (*p* < 0.05)	[88]
		Pasture + high level of concentrates (maize, sugar beet pulp, wheat bran, brewer’s yeast, dried alfalfa, barley, soybean flakes, sunflower meal, minerals and vitamins) 4.8 kg dry matter/day	Greater bitterness (*p* < 0.05) Greater solubility (*p* < 0.05) Greater firmness (*p* < 0.05) Greater granules (*p* < 0.05) More adhesive (*p* < 0.05)	
Raw Milk Caciocavallo Palermitano 1 month	Temperature 20 °C 12 Trained assessors 14 Attributes	Pasture + hay supplements (Cinisara cow breed)	More smooth texture (*p* < 0.001) More sweet taste (*p* < 0.01) More acid taste (*p* < 0.001)	[100]
		Hay + concentrate + little pasture (Brown cow breed)	More bitterness taste (*p* < 0.001) More salty taste (*p* < 0.05)	
Raw Milk Caciocavallo 90 days	12 Trained assessors 17 Attributes	Pasture (grasses, legumes and other species)	Highest yellow appearance (*p* < 0.0001) Highest spicy taste (*p* < 0.002) Highest seasoned taste (*p* < 0.003) Highest friability texture (*p* < 0.0001) Highest grainy texture (*p* < 0.0002)	[101]
		Grains (wheat, oats, barley) and hay (clover and meadow)	Highest uniform appearance (*p* < 0.0001) Highest butter odour/flavour (*p* < 0.05) Highest smoked odour/flavour (*p* < 0.002) Highest bitter taste (*p* < 0.003)	

**Table 5:** Summary of difference and consumer sensory studies on bovine cheese.

Cheese and Age	Difference Analysis	Diet	Results	Reference
Raw Milk Nostrano di Primiero 60 days	Temperature 16 °C 13 Trained assessors Triangle Test	Pasture + low level of concentrates (maize, sugar beet pulp, wheat bran, brewer’s yeast, dried alfalfa, barley, soybean flakes, sunflower meal, minerals and vitamins) 1.6 kg dry matter/day	No significant difference	[88]
		Pasture + high level of concentrates (maize, sugar beet pulp, wheat bran, brewer’s yeast, dried alfalfa, barley, soybean flakes, sunflower meal, minerals and vitamins) 4.8 kg dry matter/day	No significant difference	
**Cheese and Age**	**Consumer Analysis**	**Diet**	**Results**	**Reference**
Raw Milk St-Nectaire 8 weeks	8 Trained assessors 3 Attributes	Native mountain grassland hay + concentrate (cereal and soybean)	Highest appearance score (*p* < 0.01) Highest taste score (*p* < 0.01)	[49]
		Cocksfoot hay + concentrate (cereal and soybean)	No significant difference	
		Rye-grass rich hay + concentrate (cereal and soybean)	Highest texture score (*p* < 0.01) Highest taste score (*p* < 0.01)	
Pasteurized Cheddar 40 days	Temperature 21 °C 73–83 Untrained assessors 4 Attributes	Pasture	No significant difference	[51]
		Pasture + grain mix (soybeans, corn, sugar beet pulp and molasses)	No significant difference	
		Alfalfa hay + corn silage + concentrate (corn, grain, cottonseed, soybean meal, sugar beet pulp, yeast mix, molasses and fat)	No significant difference	
Raw Milk Caciocavallo 90 days	100 Untrained assessors	Pasture (grasses, legumes and other species)	No significant difference	[101]
		Grains (wheat, oats, barley) and hay (clover and meadow)	No significant difference	
Raw Milk Montasio 60 and 180 days	280 Untrained assessors	Mountain Pasture + concentrate (maize, barley, beet pulp, soybean and wheat) at 60 days ripening	Colour much too dark (*p* < 0.01) Colour too dark (*p* < 0.01) Holes too few (*p* < 0.05) Holes much too few (*p* < 0.01)	[50]
		Alfalfa hay, meadow hay + concentrate (maize, barley, beet pulp, soybean and wheat) at 60 days ripening	Colour about right (*p* < 0.01) Colour too light (*p* < 0.01) Holes too numerous (*p* < 0.01) Holes about right (*p* < 0.01)	
		Mountain Pasture + concentrate (maize, barley, beet pulp, soybean and wheat) at 180 days ripening	Colour much too dark (*p* < 0.01) Colour too dark (*p* < 0.01) Holes too few (*p* < 0.01) Holes much too few (*p* < 0.01)	
		Alfalfa hay, meadow hay + concentrate (maize, barley, beet pulp, soybean and wheat) at 180 days ripening	Colour about right (*p* < 0.05) Colour too light (*p* < 0.01) Holes too numerous (*p* < 0.01) Holes about right (*p* < 0.01)	
Pasteurized Milk Cheddar 180 and 270 days	Temperature 21 °C 24 Untrained assessors 5 Attributes	Pasture (rye-grass) at 180 days ripening	No significant difference	[52]
		Pasture (ryegrass) + white clover at 180 days ripening	Highest liking of appearance (*p* = 0.002) Highest overall acceptability (*p* = 0.048)	
		Concentrate (grass silage, maize silage, maize, beet pulp, soybean meal, salt, rapeseed meal and megalac) at 180 days ripening	Highest liking of texture (*p* = 0.027) Highest overall acceptability (*p* = 0.052)	
		Pasture (rye-grass) at 270 days ripening	Lowest liking of appearance (*p* = 0.013) Lowest liking of texture (*p* = 0.018)	
		Pasture (ryegrass) + white clover at 270 days ripening	No significant difference	
		Concentrate (grass silage, maize silage, maize, beet pulp, soybean meal, salt, rapeseed meal and megalac) at 270 days ripening	No significant difference	

**Table 6:** Summary of aroma analysis studies on bovine cheese.

Cheese and Age	Aroma Analysis	Diet	Results	Reference
Raw Milk St-Nectaire 8 weeks	11 Untrained assessors 13 Odour attributes	Native mountain grassland hay + concentrate (cereal and soybean)	Higher cabbage odour (*p* < 0.05)	[49]
		Cocksfoot hay + concentrate (cereal and soybean)	No significant difference	
		Rye-grass rich hay + concentrate (cereal and soybean)	No significant difference	
Raw and Pasteurized Milk Cantal-Type at 3 and 6 months	17–18 untrained assessors 9 Odour attributes	Hay + concentrate (flattened barley and soybean meal) at 3 months ripening	Higher hazelnut (*p* < 0.03)	[44]
		Raw milk cheese at 3 months ripening	Greater intensity (*p* < 0.002) Greater rancidity (*p* < 0.0005) Greater musty (*p* < 0.0001) Greater pungent (*p* < 0.0001)	
		Pasteurized milk cheese at 3 months ripening	Greater cream (*p* < 0.003) Greater hazelnut (*p* < 0.02)	
		Pasture + concentrate (flattened barley and soybean meal) at 6 months ripening	Greater musty (*p* < 0.01)	
		Hay + concentrate (flattened barley and soybean meal) at 6 months ripening	Greater butter (*p* < 0.02) Greater cream (*p* < 0.01) Greater hazelnut (*p* < 0.02)	
		Raw milk cheese at 6 months ripening	Greater intensity (*p* < 0.008) Greater rennet (*p* < 0.03) Greater musty (*p* < 0.03)	
		Pasteurized milk cheese at 6 months ripening	Greater cream (*p* < 0.009)	

## References

[1] Coulon J., Priolo A., Durand J.L., Emile J.C., Huyghe C., Lemaire G. (2002). Influence of forage feeding on the composition and organoleptic properties of meat and dairy products: Bases for a “terroir” effect. Proceedings of the 19th General Meeting of the European Grassland Federation.

[2] Martin B., Verdier-Metz I., Buchin S., Hurtaud C., Coulon J.-B. (2005). How do the nature of forages and pasture diversity influence the sensory quality of dairy livestock products?. Anim. Sci..

[3] Croissant A.E., Washburn S., Dean L., Drake M. (2007). Chemical properties and consumer perception of fluid milk from conventional and pasture-based production systems. J. Dairy Sci..

[4] Prache S. (2009). Diet authentication in sheep from the composition of animal tissues and products. Revista Brasileira de Zootecnia.

[5] Moloney A.P., Monahan F., Schmidt O. (2014). Quality and authenticity of grassland products. Future Eur. Grassl..

[6] Gaspardo B., Lavrenčič A., Levart A., Del Zotto S., Stefanon B. (2010). Use of milk fatty acids composition to discriminate area of origin of bulk milk. J. Dairy Sci..

[7] Tornambé G., Ferlay A., Farruggia A., Chilliard Y., Loiseau P., Pradel P., Graulet B., Chauveau-Duriot B., Martin B. (2010). Influence of the botanical diversity and development stage of mountain pastures on milk fatty acid composition, carotenoids, fat-soluble vitamins and sensory properties. Grassland in a Changing World, Proceedings of the 23rd General Meeting of the European Grassland Federation, Kiel, Germany, 29 August–2 September 2010.

[8] Benbrook C.M., Butler G., Latif M.A., Leifert C., Davis D.R. (2013). Organic production enhances milk nutritional quality by shifting fatty acid composition: A United States–wide, 18-month study. PLoS ONE.

[9] Larsen M.K., Kidmose U., Kristensen T., Beaumont P., Mortensen G. (2013). Chemical composition and sensory quality of bovine milk as affected by type of forage and proportion of concentrate in the feed ration. J. Sci. Food Agric..

[10] Capuano E., Van der Veer G., Boerrigter-Eenling R., Elgersma A., Rademaker J., Sterian A., Van Ruth S.M. (2014). Verification of fresh grass feeding, pasture grazing and organic farming by cows farm milk fatty acid profile. Food Chem..

[11] Hurtaud C., Dutreuil M., Coppa M., Agabriel C., Martin B. (2014). Characterization of milk from feeding systems based on herbage or corn silage with or without flaxseed and authentication through fatty acid profile. Dairy Sci. Technol..

[12] Coppa M., Chassaing C., Ferlay A., Agabriel C., Laurent C., Borreani G., Barcarolo R., Baars T., Kusche D., Harstad O.M. (2015). Potential of milk fatty acid composition to predict diet composition and authenticate feeding systems and altitude origin of European bulk milk. J. Dairy Sci..

[13] Mitani T., Kobayashi K., Ueda K., Kondo S. (2016). Discrimination of “grazing milk” using milk fatty acid profile in the grassland dairy area in Hokkaido. Anim. Sci. J..

[14] Średnicka-Tober D., Barański M., Seal C.J., Sanderson R., Benbrook C., Steinshamn H., Gromadzka-Ostrowska J., Rembiałkowska E., Skwarło-Sońta K., Eyre M. (2016). Higher PUFA and *n*-3 PUFA, conjugated linoleic acid, α-tocopherol and iron, but lower iodine and selenium concentrations in organic milk: A systematic literature review and meta-and redundancy analyses. Br. J. Nutr..

[15] Spence C. (2015). On the psychological impact of food colour. Flavour.

[16] Chilliard Y., Ferlay A. (2004). Dietary lipids and forages interactions on cow and goat milk fatty acid composition and sensory properties. Reprod. Nutr..

[17] Coulon J.-B., Delacroix-Buchet A., Martin B., Pirisi A. (2004). Relationships between ruminant management and sensory characteristics of cheeses: A review. Le Lait.

[18] Kelly M., Kolver E., Bauman D., Van Amburgh M., Muller L. (1998). Effect of Intake of Pasture on Concentrations of Conjugated Linoleic Acid in Milk of Lactating Cows. J. Dairy Sci..

[19] Chilliard Y., Glasser F., Ferlay A., Bernard L., Rouel J., Doreau M. (2007). Diet, rumen biohydrogenation and nutritional quality of cow and goat milk fat. Eur. J. Lipid Sci. Technol..

[20] Glover K., Budge S., Rose M., Rupasinghe H., MacLaren L., Green-Johnson J., Fredeen A. (2012). Effect of feeding fresh forage and marine algae on the fatty acid composition and oxidation of milk and butter. J. Dairy Sci..

[21] Rego O.A., Cabrita A.R., Rosa H.J., Alves S.P., Duarte V., Fonseca A.J., Vouzela C.F., Pires F.R., Bessa R.J. (2016). Changes in milk production and milk fatty acid composition of cows switched from pasture to a total mixed ration diet and back to pasture. Ital. J. Anim. Sci..

[22] O’Callaghan T.F., Hennessy D., McAuliffe S., Kilcawley K.N., O’Donovan M., Dillon P., Ross R.P., Stanton C. (2016). Effect of pasture versus indoor feeding systems on raw milk composition and quality over an entire lactation. J. Dairy Sci..

[23] Berry R., Hydamaka A., Noto A., Kotyk E., Zahradka P., Taylor C. (2012). Grazing period variations in cow milk vaccenic acid (VA) and conjugated linoleic acid (CLA). J. Nutr. Food Sci..

[24] Schwendel B.H., Wester T.J., Morel P.C., Fong B., Tavendale M.H., Deadman C., Shadbolt N.M., Otter D.E. (2017). Pasture feeding conventional cows removes differences between organic and conventionally produced milk. Food Chem..

[25] Palmquist D., Beaulieu A.D., Barbano D. (1993). Feed and animal factors influencing milk fat composition1. J. Dairy Sci..

[26] Kilcawley K.N., Fox P.F., Guinee T.P., Cogan T.M., McSweeney P.L.H. (2017). Cheese Flavour. Fundamentals of Cheese Science.

[27] Qian M., Burbank H., Weimer B.C. (2007). Hard Italian cheeses: Parmigiano-reggiano and grana-padano. Improving the Flavour of Cheese.

[28] Villeneuve M.-P., Lebeuf Y., Gervais R., Tremblay G., Vuillemard J., Fortin J., Chouinard P. (2013). Milk volatile organic compounds and fatty acid profile in cows fed timothy as hay, pasture, or silage. J. Dairy Sci..

[29] Li Y., Wang W. (2016). Formation of oxidized flavor compounds in concentrated milk and distillate during milk concentration. J. Dairy Sci..

[30] Zardin E., Silcock P., Siefarth C., Bremer P.J., Beauchamp J. (2016). Dynamic changes in the volatiles and sensory properties of chilled milk during exposure to light. Int. Dairy J..

[31] Havemose M.S., Weisbjerg M.R., Bredie W., Poulsen H.D., Nielsen J.H. (2006). Oxidative stability of milk influenced by fatty acids, antioxidants, and copper derived from feed. J. Dairy Sci..

[32] Hedegaard R., Kristensen D., Nielsen J.H., Frøst M.B., Østdal H., Hermansen J.E., Kröger-Ohlsen M., Skibsted L.H. (2006). Comparison of descriptive sensory analysis and chemical analysis for oxidative changes in milk. J. Dairy Sci..

[33] Romeu-Nadal M., Castellote A., López-Sabater M. (2004). Headspace gas chromatographic method for determining volatile compounds in infant formulas. J. Chromatogr. A.

[34] Bendall J.G. (2001). Aroma compounds of fresh milk from New Zealand cows fed different diets. J. Agric. Food Chem..

[35] Tunick M.H., Iandola S.K., Van Hekken D.L. (2013). Comparison of SPME methods for determining volatile compounds in milk, cheese, and whey powder. Foods.

[36] Vazquez-Landaverde P.A., Velazquez G., Torres J., Qian M. (2005). Quantitative determination of thermally derived off-flavor compounds in milk using solid-phase microextraction and gas chromatography. J. Dairy Sci..

[37] Coppa M., Ferlay A., Borreani G., Revello-Chion A., Tabacco E., Tornambé G., Pradel P., Martin B. (2015). Effect of phenological stage and proportion of fresh herbage in cow diets on milk fatty acid composition. Anim. Feed Sci. Technol..

[38] Kay J.K., Roche J.R., Kolver E.S., Thomson N.A., Baumgard L.H. (2005). A comparison between feeding systems (pasture and TMR) and the effect of vitamin E supplementation on plasma and milk fatty acid profiles in dairy cows. J. Dairy Res..

[39] Nozière P., Graulet B., Lucas A., Martin B., Grolier P., Doreau M. (2006). Carotenoids for ruminants: From forages to dairy products. Anim. Feed Sci. Technol..

[40] Kalač P. (2011). The effects of silage feeding on some sensory and health attributes of cow’s milk: A review. Food Chem..

[41] Faulkner H., O’Callaghan T.F., McAuliffe S., Hennessy D., Stanton C., O’Sullivan M.G., Kerry J.P., Kilcawley K.N. (2018). Effect of different forage types on the volatile and sensory properties of bovine milk. J. Dairy Sci..

[42] Agabriel C., Martin B., Sibra C., Bonnefoy J.-C., Montel M.-C., Didienne R., Hulin S. (2004). Effect of dairy production systems on the sensory characteristics of Cantal cheeses: A plant-scale study. Anim. Res..

[43] Forss D.A. (1979). Mechanisms of formation of aroma compounds in milk and milk products. J. Dairy Res..

[44] Cornu A., Rabiau N., Kondjoyan N., Verdier-Metz I., Pradel P., Tournayre P., Berdagué J., Martin B. (2009). Odour-active compound profiles in Cantal-type cheese: Effect of cow diet, milk pasteurization and cheese ripening. Int. Dairy J..

[45] Moorby J., Lee M., Davies D., Kim E., Nute G., Ellis N., Scollan N. (2009). Assessment of dietary ratios of red clover and grass silages on milk production and milk quality in dairy cows. J. Dairy Sci..

[46] Agabriel C., Cornu A., Journal C., Sibra C., Grolier P., Martin B. (2007). Tanker milk variability according to farm feeding practices: Vitamins A and E, carotenoids, color, and terpenoids. J. Dairy Sci..

[47] Prache S., Cornu A., Berdagué J., Priolo A. (2005). Traceability of animal feeding diet in the meat and milk of small ruminants. Small Rumin. Res..

[48] Verdier-Metz I.C., Coulon J.-B., Pradel P., Viallon C., Berdague J.-L. (1998). Effect of forage conservation (hay or silage) and cow breed on the coagulation properties of milks and on the characteristics of ripened cheeses. J. Dairy Res..

[49] Verdier-Metz I., Coulon J.-B., Pradel P., Viallon C., Albouy H., Berdagué J.-L. (2000). Effect of the botanical composition of hay and casein genetic variants on the chemical and sensory characteristics of ripened Saint-Nectaire type cheeses. Le Lait.

[50] Romanzin A., Corazzin M., Piasentier E., Bovolenta S. (2013). Effect of rearing system (mountain pasture vs. indoor) of Simmental cows on milk composition and Montasio cheese characteristics. J. Dairy Res..

[51] Khanal R., Dhiman T., Ure A., Brennand C., Boman R., McMahon D.J. (2005). Consumer acceptability of conjugated linoleic acid-enriched milk and cheddar cheese from cows grazing on pasture. J. Dairy Sci..

[52] O’Callaghan T.F., Mannion D.T., Hennessy D., McAuliffe S., O’Sullivan M.G., Leeuwendaal N., Beresford T.P., Dillon P., Kilcawley K.N., Sheehan J.J. (2017). Effect of pasture versus indoor feeding systems on quality characteristics, nutritional composition, and sensory and volatile properties of full-fat Cheddar cheese. J. Dairy Sci..

[53] Vialloninsta C., Martin B., Verdier-Metz I., Pradel P., Garel J.-P., Coulon J.-B., Berdagué J.-L. (2000). Transfer of monoterpenes and sesquiterpenes from forages into milk fat. Le Lait.

[54] Bugaud C., Buchin S., Hauwuy A., Coulon J.-B. (2001). Relationships between flavour and chemical composition of Abondance cheese derived from different types of pastures. Le Lait.

[55] Moio L., Rillo L., Ledda A., Addeo F. (1996). Odorous constituents of ovine milk in relationship to diet. J. Dairy Sci..

[56] De Feo V., Quaranta E., Fedele V., Claps S., Rubino R., Pizza C. (2006). Flavonoids and terpenoids in goat milk in relation to forage intake. Ital. J. Anim. Sci..

[57] Coppa M., Martin B., Pradel P., Leotta B., Priolo A., Vasta V. (2011). Effect of a hay-based diet or different upland grazing systems on milk volatile compounds. J. Agric. Food Chem..

[58] Fernandez C., Astier C., Rock E., Coulon J.B., Berdagué J.L. (2003). Characterization of milk by analysis of its terpene fractions. Int. J. Food Sci. Technol..

[59] Lejonklev J., Løkke M.M., Larsen M.K., Mortensen G., Petersen M.A., Weisbjerg M.R. (2013). Transfer of terpenes from essential oils into cow milk. J. Dairy Sci..

[60] Tornambé G., Cornu A., Pradel P., Kondjoyan N., Carnat A., Petit M., Martin B. (2006). Changes in terpene content in milk from pasture-fed cows. J. Dairy Sci..

[61] Engel E., Ferlay A., Cornu A., Chilliard Y., Agabriel C., Bielicki G., Martin B. (2007). Relevance of isotopic and molecular biomarkers for the authentication of milk according to production zone and type of feeding of the cow. J. Agric. Food Chem..

[62] Viallon C.V.-M., Verdier-Metz I., Denoyer C., Pradel P., Coulon J.-B., Berdague J.-L. (1999). Desorbed terpenes and sesquiterpenes from forages and cheeses. J. Dairy Res..

[63] Carpino S., Mallia S., La Terra S., Melilli C., Licitra G., Acree T., Barbano D., Van Soest P. (2004). Composition and aroma compounds of Ragusano cheese: Native pasture and total mixed rations. J. Dairy Sci..

[64] De Noni I., Battelli G. (2008). Terpenes and fatty acid profiles of milk fat and “Bitto” cheese as affected by transhumance of cows on different mountain pastures. Food Chem..

[65] O’Callaghan T.F., Faulkner H., McAuliffe S., O’Sullivan M.G., Hennessy D., Dillon P., Kilcawley K.N., Stanton C., Ross R.P. (2016). Quality characteristics, chemical composition, and sensory properties of butter from cows on pasture versus indoor feeding systems. J. Dairy Sci..

[66] Chion A.R., Tabacco E., Giaccone D., Peiretti P.G., Battelli G., Borreani G. (2010). Variation of fatty acid and terpene profiles in mountain milk and “Toma piemontese” cheese as affected by diet composition in different seasons. Food Chem..

[67] Toso B., Procida G., Stefanon B. (2002). Determination of volatile compounds in cows’ milk using headspace GC-MS. J. Dairy Res..

[68] Figueiredo R., Rodrigues A.I., do Céu Costa M. (2007). Volatile composition of red clover (*Trifolium pratense* L.) forages in Portugal: The influence of ripening stage and ensilage. Food Chem..

[69] Boltar I., Majhenič A.Č., Jarni K., Jug T., Kralj M.B. (2015). Volatile compounds in Nanos cheese: Their formation during ripening and sesonal variation. J. Food Sci. Technol..

[70] Bovolenta S., Romanzin A., Corazzin M., Spanghero M., Aprea E., Gasperi F., Piasentier E. (2014). Volatile compounds and sensory properties of Montasio cheese made from the milk of Simmental cows grazing on alpine pastures. J. Dairy Sci..

[71] Buchin S., Martin B., Dupont D., Bornard A., Achilleos C. (1999). Influence of the composition of Alpine highland pasture on the chemical, rheological and sensory properties of cheese. J. Dairy Res..

[72] Poulopoulou I., Zoidis E., Massouras T., Hadjigeorgiou I. (2012). Terpenes transfer to milk and cheese after oral administration to sheep fed indoors. J. Anim. Physiol. Anim. Nutr..

[73] Schlichtherle-Cerny H., Imhof M., Fernández García E., Bosset J. (2004). Changes in terpene composition from pasture to cheese. Mitteilungen aus Lebensmitteluntersuchung und Hygiene.

[74] Belviso S., Giordano M., Dolci P., Zeppa G. (2011). Degradation and biosynthesis of terpenoids by lactic acid bacteria isolated from cheese: First evidence. Dairy Sci. Technol..

[75] Stefanon B., Procida G. (2004). Effects of including silage in the diet on volatile compound profiles in Montasio cheese and their modification during ripening. J. Dairy Res..

[76] Bendall J.G., Olney S.D. (2001). Hept-*cis*-4-enal: Analysis and flavour contribution to fresh milk. Int. Dairy J..

[77] Mounchili A., Wichtel J., Bosset J., Dohoo I.R., Imhof M., Altieri D., Mallia S., Stryhn H. (2005). HS-SPME gas chromatographic characterization of volatile compounds in milk tainted with off-flavour. Int. Dairy J..

[78] Falchero L., Lombardi G., Gorlier A., Lonati M., Odoardi M., Cavallero A. (2010). Variation in fatty acid composition of milk and cheese from cows grazed on two alpine pastures. Dairy Sci. Technol..

[79] Urbach G. (1997). The flavour of milk and dairy products: II. Cheese: Contribution of volatile compounds. Int. J. Dairy Technol..

[80] Aprea E., Romanzin A., Corazzin M., Favotto S., Betta E., Gasperi F., Bovolenta S. (2016). Effects of grazing cow diet on volatile compounds as well as physicochemical and sensory characteristics of 12-month-ripened Montasio cheese. J. Dairy Sci..

[81] Kilic M., Lindsay R. (2005). Distribution of conjugates of alkylphenols in milk from different ruminant species. J. Dairy Sci..

[82] Lopez V., Lindsay R.C. (1993). Metabolic conjugates as precursors for characterizing flavor compounds in ruminant milks. J. Agric. Food Chem..

[83] Guichard G., Leconte D., Picoche B., Simon J.-C. (2006). Influence de la composition floristique des prairies permanentes normandes sur les caractéristiques des laits crus dérivés. Fourrages.

[84] Dubroeucq H., Martin B., Ferlay A., Pradel P., Verdier-Metz I., Chilliard Y., Agabriel J., Coulon J. (2002). Cow’s feeding may modify sensory properties of milk. Rencontres Rech. Rumin..

[85] Verdier-Metz I., Martin B., Pradel P., Albouy H., Hulin S., Montel M.-C., Coulon J.-B. (2005). Effect of grass-silage vs. hay diet on the characteristics of cheese: Interactions with the cheese model. Le Lait.

[86] Tornambé G., Cornu A., Verdier-Metz I., Pradel P., Kondjoyan N., Figueredo G., Hulin S., Martin B. (2008). Addition of pasture plant essential oil in milk: Influence on chemical and sensory properties of milk and cheese. J. Dairy Sci..

[87] Lerch S., Ferlay A., Graulet B., Cirié C., Verdier-Metz I., Montel M., Chilliard Y., Martin B. (2015). Extruded linseeds, vitamin E and plant extracts in corn silage-based diets of dairy cows: Effects on sensory properties of raw milk and uncooked pressed cheese. Int. Dairy J..

[88] Bovolenta S., Corazzin M., Saccà E., Gasperi F., Biasioli F., Ventura W. (2009). Performance and cheese quality of Brown cows grazing on mountain pasture fed two different levels of supplementation. Livest. Sci..

[89] Bertilsson J., Murphy M. (2003). Effects of feeding clover silages on feed intake, milk production and digestion in dairy cows. Grass Forage Sci..

[90] Hougaard A.B., Vestergaard J.S., Varming C., Bredie W.L., Ipsen R.H. (2011). Composition of volatile compounds in bovine milk heat treated by instant infusion pasteurisation and their correlation to sensory analysis. Int. J. Dairy Technol..

[91] Contarini G., Povolo M., Leardi R., Toppino P.M. (1997). Influence of heat treatment on the volatile compounds of milk. J. Agric. Food Chem..

[92] Zepka L.Q., Garruti D.S., Sampaio K.L., Mercadante A.Z., Da Silva M.A.A. (2014). Aroma compounds derived from the thermal degradation of carotenoids in a cashew apple juice model. Food Res. Int..

[93] Calvo M.M., de la Hoz L. (1992). Flavour of heated milks. A review. Int. Dairy J..

[94] Mogensen L., Vestergaard J.S., Fretté X., Lund P., Weisbjerg M.R., Kristensen T. (2010). Effect of toasting field beans and of grass-clover: Maize silage ratio on milk production, milk composition and sensory quality of milk. Livest. Sci..

[95] Lejonklev J., Kidmose U., Jensen S., Petersen M.A., Helwing A., Mortensen G., Weisbjerg M.R., Larsen M.K. (2016). Effect of oregano and caraway essential oils on the production and flavor of cow milk. J. Dairy Sci..

[96] Shingfield K.J., Salo-Väänänen P., Pahkala E., Toivonen V., Jaakkola S., Piironen V., Huhtanen P. (2005). Effect of forage conservation method, concentrate level and propylene glycol on the fatty acid composition and vitamin content of cows’ milk. J. Dairy Res..

[97] Gaillard C., Sørensen M.T., Vestergaard M., Weisbjerg M.R., Basar A., Larsen M.K., Martinussen H., Kidmose U., Sehested J. (2017). Effect of substituting soybean meal and canola cake with dried distillers grains with solubles at 2 dietary crude protein levels on feed intake, milk production, and milk quality in dairy cows. J. Dairy Sci..

[98] Yayota M., Tsukamoto M., Yamada Y., Ohtani S. (2013). Milk composition and flavor under different feeding systems: A survey of dairy farms. J. Dairy Sci..

[99] Carpino S., Horne J., Melilli C., Licitra G., Barbano D., Van Soest P. (2004). Contribution of native pasture to the sensory properties of Ragusano cheese. J. Dairy Sci..

[100] Bonanno A., Tornambè G., Bellina V., De Pasquale C., Mazza F., Maniaci G., Di Grigoli A. (2013). Effect of farming system and cheesemaking technology on the physicochemical characteristics, fatty acid profile, and sensory properties of Caciocavallo Palermitano cheese. J. Dairy Sci..

[101] Esposito G., Masucci F., Napolitano F., Braghieri A., Romano R., Manzo N., Di Francia A. (2014). Fatty acid and sensory profiles of Caciocavallo cheese as affected by management system. J. Dairy Sci..

